# Growth differentiation factor 11 attenuates cardiac ischemia reperfusion injury via enhancing mitochondrial biogenesis and telomerase activity

**DOI:** 10.1038/s41419-021-03954-8

**Published:** 2021-07-02

**Authors:** Lin Chen, Guangjin Luo, Yameng Liu, Hairuo Lin, Cankun Zheng, Dongxiao Xie, Yingqi Zhu, Lu Chen, Xiaoxia Huang, Donghong Hu, Jiahe Xie, Zhenhuan Chen, Wangjun Liao, Jianping Bin, Qiancheng Wang, Yulin Liao

**Affiliations:** 1 Department of Cardiology, State Key Laboratory of Organ Failure Research, Guangdong Provincial Key Laboratory of Shock and Microcirculation, Nanfang Hospital, Southern Medical University, Guangzhou, 510515 China; 2 Department of Oncology, Nanfang Hospital, Southern Medical University, Guangzhou, Guangdong 510515 China; 3 National Clinical Research Center of Kidney Disease, Guangdong Provincial Institute of Nephrology, Nanfang Hospital, Southern Medical University, Guangzhou, 510515 China

**Keywords:** Apoptosis, Heart failure

## Abstract

It has been reported that growth differentiation factor 11 (GDF11) protects against myocardial ischemia/reperfusion (IR) injury, but the underlying mechanisms have not been fully clarified. Considering that GDF11 plays a role in the aging/rejuvenation process and that aging is associated with telomere shortening and cardiac dysfunction, we hypothesized that GDF11 might protect against IR injury by activating telomerase. Human plasma GDF11 levels were significantly lower in acute coronary syndrome patients than in chronic coronary syndrome patients. IR mice with myocardial overexpression GDF11 (oe-GDF11) exhibited a significantly smaller myocardial infarct size, less cardiac remodeling and dysfunction, fewer apoptotic cardiomyocytes, higher telomerase activity, longer telomeres, and higher ATP generation than IR mice treated with an adenovirus carrying a negative control plasmid. Furthermore, mitochondrial biogenesis-related proteins and some antiapoptotic proteins were significantly upregulated by oe-GDF11. These cardioprotective effects of oe-GDF11 were significantly antagonized by BIBR1532, a specific telomerase inhibitor. Similar effects of oe-GDF11 on apoptosis and mitochondrial energy biogenesis were observed in cultured neonatal rat cardiomyocytes, whereas GDF11 silencing elicited the opposite effects to oe-GDF11 in mice. We concluded that telomerase activation by GDF11 contributes to the alleviation of myocardial IR injury through enhancing mitochondrial biogenesis and suppressing cardiomyocyte apoptosis.

## Introduction

Growth differentiation factor 11 (GDF11) has attracted much attention as a potential antiaging candidate [1]. However, its role in rejuvenation is controversial [2, 3]. Initial studies in rodent models revealed that increasing the protein levels of GDF11 in aged mice improves age-related phenotypes in the brain, heart, and skeletal muscle [4–6], but subsequently, investigators have questioned the beneficial role of circulating GDF11, reporting that GDF11 levels increase with age and cause muscle atrophy and cachexia rather than fostering rejuvenation [3, 7]. Many factors, such as the dose used, the protein expression levels of GDF11, the experimental design, and the reagents used to detect GDF11, are thought to have contributed to these conflicting results [8]. Therefore, additional work is necessary to elucidate the antiaging effects of GDF11. Intriguingly, accumulating evidence suggests that GDF11 is cardioprotective in various pathological states. Deletion of GDF11 in cardiomyocytes leads to left ventricular dilation [9], while exogenous recombinant GDF11 can alleviate diet-induced weight gain and improve metabolic homeostasis [10]. Although GDF11 has been suggested to have an antihypertrophic effect in aging mice and patients [6, 11], the effects of endogenous GDF11 on myocardial ischemia or ischemia/reperfusion (IR) as well as its underlying mechanisms have not been systematically investigated.

To date, only a few reports have demonstrated the cardioprotective effects of GDF11 on myocardial ischemia. In patients with stable ischemic heart disease, higher GDF11/8 levels have been reported to be associated with a lower risk of cardiovascular events and death [11], while both endogenous and exogenous GDF11 have been demonstrated to protect the heart against IR injury in mice by enhancing the proliferation of cardiac progenitor cells [12] and attenuating noncanonical transforming growth factor-β (TGF-β) signaling, respectively. Additional evidence and clarification of the potential mechanisms underlying the role of GDF11 in myocardial ischemia would be helpful for identifying new therapeutic targets.

Telomeres are heterochromatic structures at the ends of linear chromosomes that protect them from degradation and DNA repair activities, and are essential to ensure chromosome stability. Mammalian telomeres comprise several kilobases of tandem TTAGGG DNA repeats [13]. Telomere shortening is a well-established cause of cellular senescence and apoptosis that can be induced by many factors, and telomere length (TL) is positively related to the life span of cells [14]. Early studies have shown TL is maintained by the telomerase [15], a complex of ribonucleoproteins containing two core components, a catalytic telomerase reverse transcriptase (TERT) and a telomerase RNA component (Terc) [16]. Activation of TERT is implicated in the synthesis of new telomeric DNA repeats, thereby overcoming telomeric DNA attrition from the ends of the chromosomes [14]. Clinical investigations have shown that a shorter blood leukocyte TL is related to worse cardiovascular outcomes after acute myocardial infarction or an increased risk of myocardial infarction in patients [17, 18], while transplantation of cardiosphere-derived cells to the hearts of aging mice neither increases TL nor improves cardiac function [19]. Although leukocyte TL is highly correlated with TL in various somatic tissues, little direct evidence is available on whether cardiomyocyte TL is a therapeutic target for myocardial ischemia.

Considering that both GDF11 and TL are closely associated with aging [20], we are curious whether GDF11 can protect the heart by increasing TL. GDF11 can inhibit myocardial oxidative stress occurs in mitochondria [21], while impaired TL and low telomerase activity are related to mitochondrial dysfunction and resulting sustained p53 activation [22, 23] and consequently result in cellular apoptosis [24]. Accordingly, we hypothesized that activation of telomerase might contribute to the cardioprotective effects of GDF11 in IR injury through enhancing mitochondrial biogenesis and suppressing cardiomyocyte apoptosis.

## Materials and methods

### Participants and measurement of plasma GDF11

The cohort consisted of patients who were diagnosed with coronary artery disease (CAD) and healthy participants collected from Nanfang Hospital between January 2018 and September 2018. Patients with CAD were divided into the chronic coronary syndrome (CCS) group and acute coronary syndrome (ACS) group according to diagnostic criteria [25]. Clinical characteristics and flow of study participants are summarized in Supplementary Table S1 and Fig. S1. We excluded patients if they (1) were younger than 15 years, (2) had a history of other cardiac diseases, including valvular disease and cardiomyopathy, or (3) had a history of severe systemic diseases, such as malignancies, serious infection, and hepatic disease. Hence, the final cohort included 218 participants. Human plasma concentrations of GDF11, cardiac troponin I (cTn-I) and mouse plasma concentration of GDF11 were determined using the CSB-EL009344HU (Cusabio), CSB-E05139h ELISA kits (Cusabio) and KE1559 ELISA kit (ImmunoWay) respectively, according to the manufacturers’ instructions.

### IR model generation, infarct size, and histological examination in mice

C57/BL6 male mice aged 10 weeks were randomly assigned to sham group and IR group and were anesthetized with a mixture of ketamine (100 mg/kg, intraperitoneal) and xylazine (5 mg/kg, intraperitoneal), the left coronary artery (LCA) was ligated for 45 min, and then the ligation was removed to allow reperfusion for 24 h or 28 days after surgery. Sham-operated mice underwent an identical surgical operation without ligation. Myocardial ischemia was evaluated based on ST segment elevation on electrocardiogram (ECG). The animals were killed by 2% isoflurane inhalation and cervical dislocation 24 h or 28 days after surgery to observe the short-term and long-term effects of GDF11 on IR injury (Fig. S2).

Twenty-four hours after surgery, the hearts of mice were harvested and cut into pieces. Myocardial infarction was confirmed by staining the tissue with 1% triphenyl tetrazolium chloride (TTC) (Sigma Aldrich, USA) at 37 °C for 20 min, and the area at risk (AAR) was determined by aortic injection of Evans blue. Myocardial infarct size (IS) was measured using Image J software [26].

Twenty-eight days after surgery, the hearts were excised, rinsed with phosphate-buffered saline (PBS), fixed in 4% paraformaldehyde and embedded in paraffin; 4–6 μm sections were prepared. Masson’s trichrome staining was utilized to evaluate myocardial fibrosis. For troponin T staining, paraffin sections obtained as described above were rinsed three times in PBS, blocked in buffer (10% bovine serum albumin; BSA; 20 min), and rinsed three times in PBS. The slides were incubated (4 °C) overnight with primary antibody against cardiac troponin T (1:100, mouse monoclonal, Santa Cruz, CA, USA). The slides were rinsed in PBS three times the following day and incubated with secondary antibodies conjugated to Alexa Fluor 488 or 555 (1:100 dilution; Santa Cruz, CA, USA) for 1 h at room temperature. The slides were washed three times in PBS and stained with DAPI for 3 min to label nuclei.

### Anoxia/reoxygenation (AR) of neonatal rat cardiomyocytes (NRCMs) and cell viability assay

NRCMs were harvested as described elsewhere [27]. The cells were placed in either a low-oxygen atmosphere or normoxic conditions. Normoxic conditions of 5% CO_2_ and 21% O_2_ were created at 37 °C in a normoxic incubator. To induce anoxia, the NRCMs were cultured in a humidified condition at 37 °C in a hypoxic chamber maintained at 5% CO_2_ and 1% O_2_ for 3 h. After anoxia, the NRCMs were cultured under normoxic conditions for 2 h reoxygenation. The cells incubated under normoxic conditions that were not subjected to AR and served as a control. The cell viability was determined using the MTT assay (ab211091, Abcam, Cambridge, UK) based on the manufacturer’s instructions.

### Transmission election microscopy (TEM)

Tissue of mouse heart was fixed with 3% glutaraldehyde, rinsed with cacodylate buffer, and postfixed with 1% osmium tetroxide in 0.1 M SC buffer for 1 h. After rinse, the tissue specimens were dehydrated through graded ethyl alcohols and then infiltrated with 100% acetone and embedding resin for 2 days. Following polymerization at 60 °C overnight, the blocks were then sections. Thin sections were cut and stained with UA replacement stain. The mitochondrial content was determined by quantifying the number and size of each mitochondrion per field using Image J software [28]. Morphometric analysis of distances between cristae was performed in ten randomly selected mitochondria of each cell, fifty cells per sample at least. The distances between cristae were quantified using the multimeasure and ROI plugins of Image J software [29]. Average distance between cristae in each mitochondrial was presented as mean ± SD of five independent experiments.

### Construction of adenovirus carrying a GDF11 or short hairpin-GDF11 plasmid

A CMV-MCS-EGFP vector carrying the adenovirus (Ad)-GDF11 plasmid (oe-GDF11), HU6-MCS-CMV-EGFP vectors carrying the Ad-GDF11-shRNA (short hairpin RNA; sh-1:5′-GCCTGAGGACTTCTTGGAA-3′; sh-2: 5′-GCAGATCTTACGACTGAAA-3′; sh-3: 5′-GCCGATATCCTCTCACAGT-3′) plasmid (sh-GDF11) and negative controls (NC) (CMV-MCS-3FLAG-SV40-EGFP vector carrying empty plasmid) were generated by a commercial company (Genechem Company, Shanghai, China). For in vivo infection, ad particles were multipoint injected into the left ventricles of the mice 72 h before IR surgery. Oe-GDF11, sh-GDF11, and NC virus particles (1 × 10^11^ viral genomes/ml) were administered by using a 300 μl syringe with 30 gauge needle to direct inject into the LV free wall (three evenly dispersed sites, 10 μl/site) in 8-week-old mice. Do not insert the syringe needle too deep to avoid piercing the ventricular wall muscles into the LV [30]. Twenty-four hours after IR or sham, the samples were harvested for total protein or mRNA extraction. For in vitro infection, the recombinant Ad were directly transfected 3 days before AR (multiplicity of infection = 200) in cultured NRCMs. The recombinant Ad transduction efficiency was evaluated by fluorescence microscopy with EGFP fluorescence (510 nm). Transfected cells were harvested for total protein or mRNA extraction after AR.

### Real-time quantitative polymerase chain reaction (PCR) and western blotting

Total RNA was extracted from heart tissues using TRIzol reagent (Invitrogen, USA). Real-time qPCR was performed using a Quantitect SYBR RT-PCR kit (DRR420A, Takara, Japan) to evaluate mRNA levels in heart tissues. The primer sequences are listed in Table S2.

Proteins were obtained from NRCMs or whole-heart homogenates. Samples were separated by 10% sodium dodecyl sulfate-polyacrylamide gel electrophoresis. The proteins were transferred onto polyvinyl difluoride membranes. 5% BSA was used to block the membranes for 2 h. Primary antibody were incubated at 4 °C overnight. The following antibodies were used for western blotting: anti-GDF11 (ab124721, Abcam, Cambridge, UK), anti-GAPDH (#2118, CST, MA, USA), anti-PGC-1α (ab54481, Abcam, Cambridge, UK), anti-TFAM (#7495, CST, MA, USA), anti-Bax (#14796, CST, MA, USA), anti-P53 (#2524, CST, MA, USA), anti-p-P53 (#12571, Ser15, CST, MA, USA), anti-PI3K (#4257, CST, MA, USA), anti-Akt (#9272, CST, MA, USA), anti-p-Akt (#9271, Ser473, CST, MA, USA), anti-FoxO3a (#12829, CST, MA, USA), anti-Bcl2 (#sc7382, Santa Cruz, CA, USA), anti-Caspase3 (#9662S, CST, MA, USA), and anti-Cleaved-caspase3 (C-caspase3, #9661S, CST, MA, USA) antibodies. The bands were detected using the Super Signal ECL Kit (Invitrogen, Carlsbad, CA, USA) on a Western blotting detection system (Kodak Digital Science, Rochester, NY, USA) and quantified by densitometry using Image J software.

### Measurement of TL and telomerase activity

#### PCR method for TL

DNA samples were extracted from mouse heart tissues and NRCMs with the PureLink™ Genomic DNA Mini Kit (k1820-01, Invitrogen). The mean TL of cardiomyocytes was assessed by a modified monochrome multiplex quantitative PCR method. The relative TL is shown as the *T*/*S* ratio (the ratio of telomere repeat copy number to single-copy gene copy number) [31]. All primers used in this study are listed in Table S2.

#### Q-FISH method for TL

After deparaffinization, tissues were postfixed in 4% formaldehyde. Tissues were incubated at 37 °C for 15 min in pepsin solution (Sigma Aldrich). The tissue sections were mounted on slides and dehydrated in ethanol. After 10 min of air drying, 0.5 mg/ml PNA probe (Panagene) were added to each slide. The slides were incubated for 3 min at 90 °C and for an additional 2 h at room temperature in the dark. Then, the slides were incubated with DAPI (Sigma Aldrich). Confocal images were acquired as stacks using a Leica SP5-MP confocal microscope and maximum projections were done with the LAS-AF software. Telomere signal intensity was quantified using Definiens software. Fifty images per sample were captured. TL values were analyzed using individual telomere spots (300,000 telomere spots per sample). The average fluorescence intensities represent the TL of each sample [15].

Telomerase activity was quantified with a modified fluorescence telomere repeat amplification assay according to the protocol of the Telomerase Activity Quantification qPCR Assay Kit (KGA1028R, Keygen).

### Measurement of mitochondrial energy dynamics

#### ATP measurement

The level of ATP was detected using an ATP assay kit (KA1661, Abnova) based on the manufacturer’s instructions. NRCMs in 6-well plates were lysed and centrifuged at 12000 × *g* for 5 min at 4 °C. Subsequently, supernatant was mixed with luciferase reagent in an opaque 96-well plate and measured using a chemiluminometer.

#### Oxygen consumption

The level of oxygen consumption was detected using an oxygen consumption kit (BB48211, Bestbio) based on the manufacturer’s instructions.

#### Mitochondrial DNA (mtDNA)

mtDNA was extracted using a mitochondrial DNA isolation kit (K280-50, Biovision) according to the company’s recommended protocol. The mtDNA copy number was measured using a mtDNA PCR Kit based on the manufacturer’s instructions (VH01006, VIPOTION). The ratio of mtDNA to 18S rRNA was calculated and served as the mtDNA copy number for each group.

#### Mitochondrial protein content

Tissue or cell mitochondrial proteins were extracted using a cytoplasmic and mitochondrial protein extraction kit (C7610, Solarbio) and tissue or cell total proteins were extracted using a total protein extraction kit (BC3711, Solarbio), according to the company’s recommended protocols. Tissue samples (200 mg) were taken from the border area of the left ventricular infarction. The mitochondrial protein and total protein concentrations were quantified after extraction. The ratio of mitochondrial protein/total protein represented the relative content of mitochondrial proteins.

### Apoptosis determination

Apoptosis was measured by terminal deoxyribonucleotidyl transferase-mediated TdT-mediated dUTP nick end labeling (TUNEL) with a kit based on the company’s protocol (Roche, Germany). The percentage of apoptotic cells was calculated as the ratio of TUNEL-positive cells to total nuclei.

### Statistical analysis

Quantitative data are expressed as mean (±SD). Comparisons of two groups were performed by Student’s unpaired two-tailed *t*-test; and comparisons of parameters among ≥3 groups, by one-way ANOVA followed by Bonferroni’s correction for post-hoc multiple comparisons. Test of variance homogeneity was performed before analysis between the groups that are being statistically compared. Linear correlations between selected variables were assessed by the least-squares method. Kaplan–Meier survival analysis was used to evaluate the overall survival of mice for 4 weeks and the groups were compared by the log-rank test. All analyses were performed with GraphPad Prism 7.0 software (GraphPad Software Inc., San Diego, CA, USA), and *P* < 0.05 was considered to be statistically significant.

## Results

### GDF11 is decreased in patients with CAD and in mice subjected to myocardial IR

**Fig. 1:**
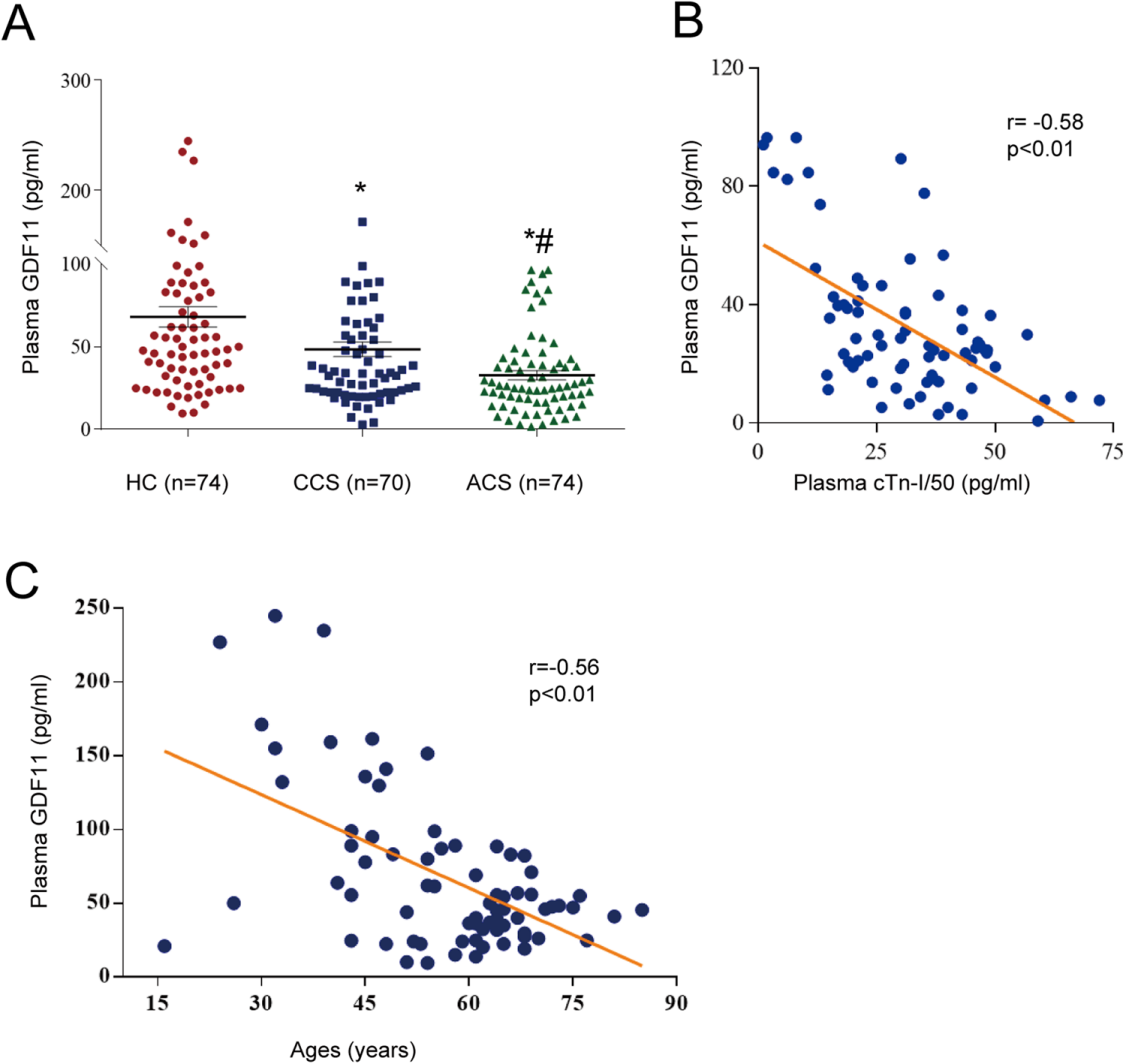
Plasma GDF11 levels in patients with CAD. **A** Plasma GDF11 levels in the healthy control (HC) group (*n* = 74), patients with chronic coronary syndrome (CCS) (*n* = 70) and patients with acute coronary syndrome (ACS) (*n* = 74); *p < 0.05 vs. the HC group; ^#^*p* < 0.05 vs. the CCS group; one-way ANOVA with Tukey’s multiple comparisons test. **B** Correlation between plasma GDF11 levels and plasma cTn-I levels in the ACS group; *p* < 0.01, Pearson correlations. **C** Correlation between plasma GDF11 levels and age in the non-CAD group; *p* < 0.01, Pearson correlations. GDF11 growth differentiation factor 11, cTn-I cardiac troponin I, CAD coronary artery disease.

The plasma concentrations of GDF11 in both the CCS and ACS groups were significantly lower than those in the healthy control group (*p* < 0.01, Fig. 1A) and were lower in the ACS group than in the CCS group (*p* < 0.01, Fig. 1A). Significant negative correlations were found between plasma GDF11 and troponin-I levels in the ACS group, and between plasma GDF11 levels and age in the control group (*r* = −0.58 and 0.56, respectively, both *p* < 0.01, Fig. 1B, C).

**Fig. 2:**
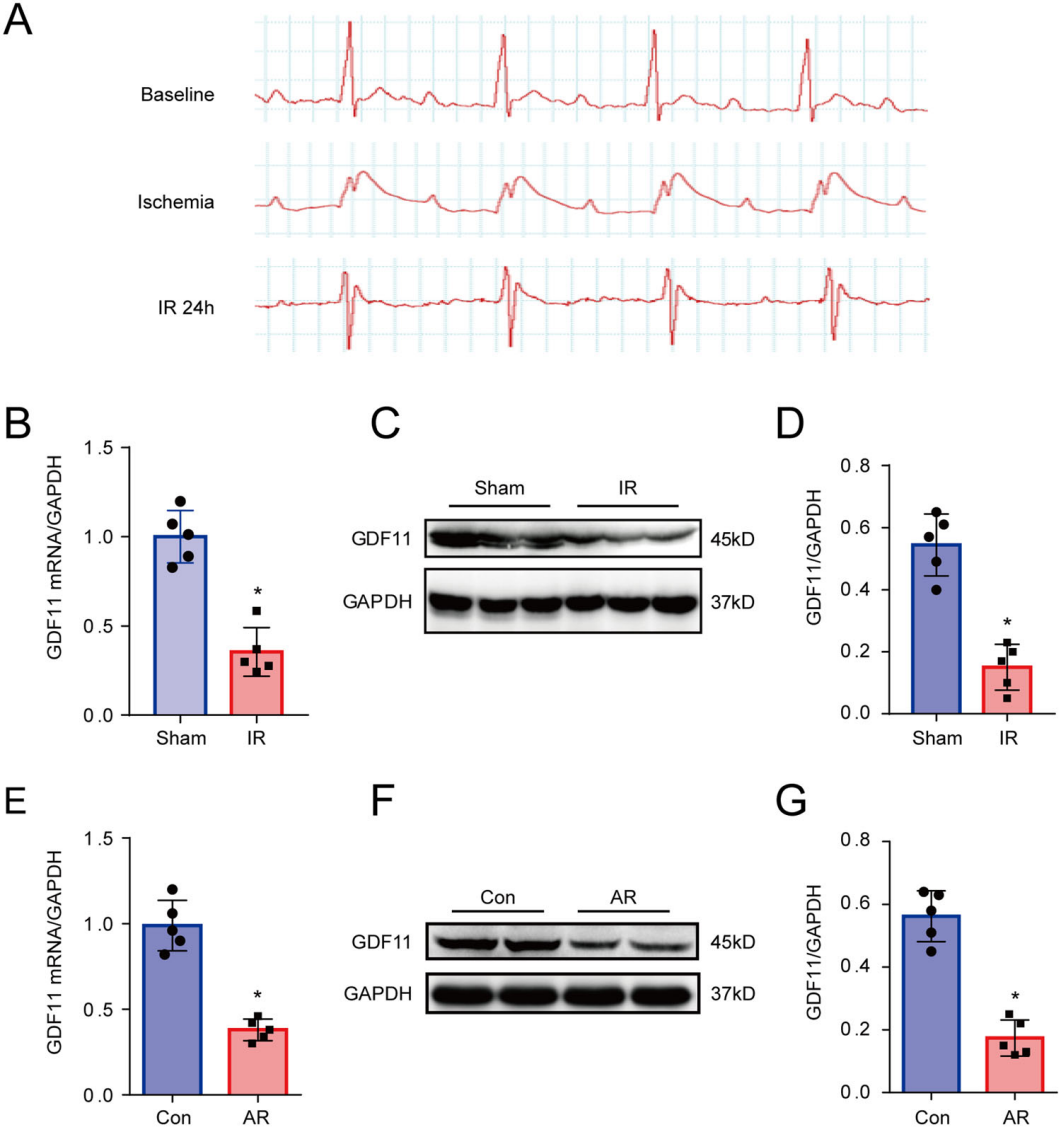
GDF11 was downregulated in rodent cardiomyocytes following IR or AR. **A** Changes in the ST segment on ECG in response to myocardial IR in mice. **B** GDF11 mRNA levels in sham and IR mice at 24 h after surgery. **C** Representative western blots of GDF11 in sham and IR groups. **D** GDF11 protein levels in sham and IR mice at 24 h after surgery. **E** GDF11 mRNA levels in NRCMs subjected to AR. **F** Representative western blots of GDF11 in NRCMs subjected to AR. **G** GDF11 protein levels in NRCMs subjected to AR. *p < 0.05 vs. the sham group, *n* = 5 in each group. GDF11 growth differentiation factor 11, ECG electrocardiogram, IR ischemia/reperfusion, AR anoxia/reoxygenation, NRCMs neonatal rat cardiomyocytes.

In mice subjected to IR (Fig. 2), myocardial GDF11 mRNA and protein levels were significantly lower than those in the sham group (Fig. 2B–D). Similar results were obtained in cultured NRCMs subjected to AR (Fig. 2E–G).

### Oe-GDF11 reduces myocardial IS and attenuates cardiac remodeling

To further investigate the influence of GDF11 on IR injury, we constructed oe-GDF11, sh-GDF11, or negative control (NC) adenovirus particles. For in vivo infection, we employed multipoint myocardial injection of adenoviruses to overexpress and knockdown GDF11 in the heart, which prevented GDF11 from impacting other organs, such as liver, spleen, lung, kidney, and skeletal muscle. Compared with the NC group, there was no significant difference in GDF11 mRNA expressions in other organs, morphological analysis, organ weight/body weight ratio, or body weight in oe-GDF11-treated mice and sh-GDF11-treated mice. (Fig. S3A–D). In addition, the data on plasma GDF11 levels in mice treated with oe-GDF11/sh-GDF11 Ad particles showed that plasma GDF11 levels were increased/decreased by approximately 1.3-fold and 0.8-fold, respectively (Fig. S3E).

For in vitro infection, the adenoviruses were directly transfected 3 days before AR (multiplicity of infection = 200) in NRCMs. In addition, we confirmed the gene and protein expression levels of GDF11 in the mouse heart and NRCMs (Fig. S4A–L). The protein levels of GDF11 were increased by approximately 2-fold and decreased by approximately 60–70% in response to treatment with oe-GDF11 and sh-GDF11, respectively (Fig. S4C, F, I, and L).

**Fig. 3:**
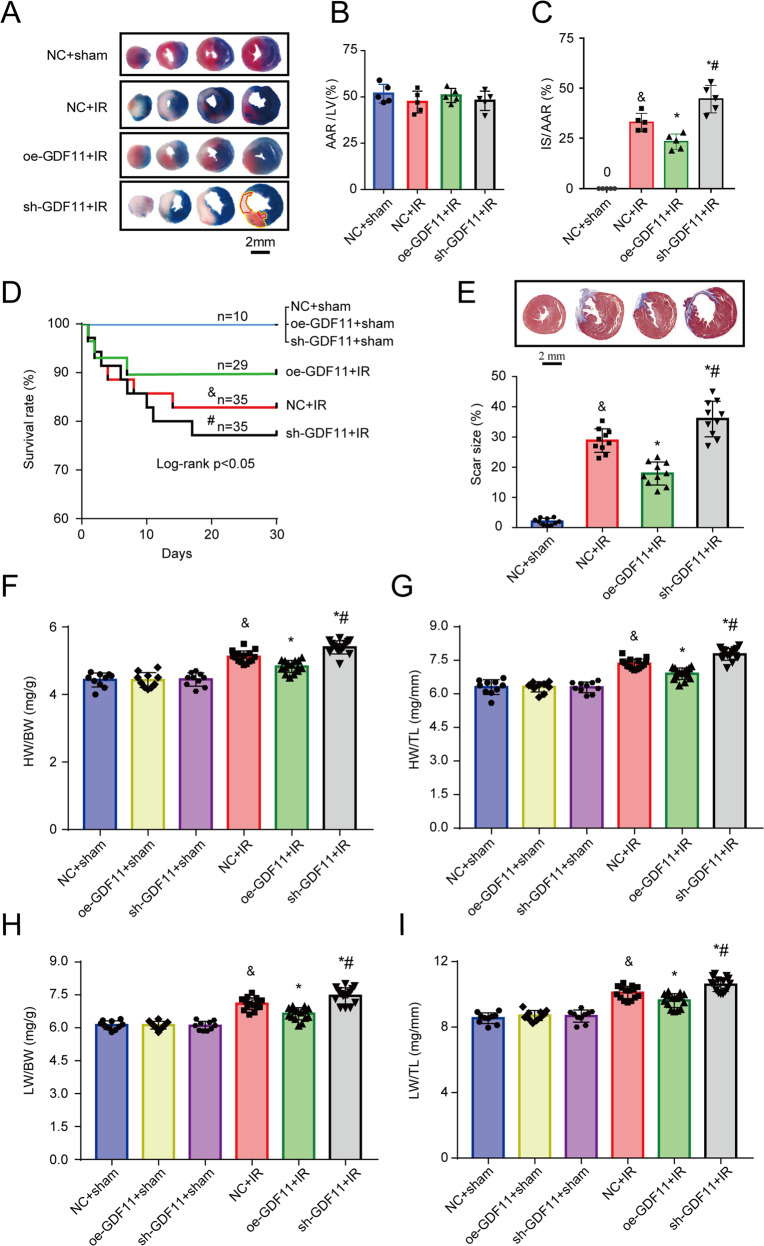
Effects of GDF11 on the myocardial IS, survival and cardiac remodeling in mice subjected to IR. Adenovirus carrying GDF11 (oe-GDF11) or adenovirus carrying sh-GDF11 were injected into the myocardium. **A** Representative pictures of Evans blue and triphenyl tetrazolium chloride (TTC) double-stained myocardial sections from mice subjected to 45 min of ischemia/24 h of reperfusion. Scale bar = 2 mm. **B** AAR in the four groups at 24 h after surgery; *n* = 5, one-way ANOVA. **C** Myocardial IS in the four groups at 24 h after surgery; *n* = 5, one-way ANOVA followed by Bonferroni’s correction for post hoc multiple comparisons. **D** Four-week survival curves of the six groups. ^&^*p* < 0.05 vs. the sham group; ^#^*p* < 0.05 vs. the oe-GDF11 + IR group, Kaplan–Meier survival analysis. **E** Myocardial scar size at 4 weeks after surgery; *n* = 10, one-way ANOVA followed by Bonferroni’s correction for post hoc multiple comparisons. **F** The heart weight (HW)/body weight (BW) ratio at 4 weeks after surgery; *n* = 10–15, one-way ANOVA followed by Bonferroni’s correction for post-hoc multiple comparisons. **G** The HW/TiL ratio at 4 weeks after surgery; *n* = 10–15, one-way ANOVA followed by Bonferroni’s correction for post hoc multiple comparisons. **H** The lung weight (LW)/BW ratio at 4 weeks after surgery; *n* = 10–15, one-way ANOVA followed by Bonferroni’s correction for post-hoc multiple comparisons. **I** The LW/TiL ratio at 4 weeks after surgery; *n* = 10–15, one-way ANOVA followed by Bonferroni’s correction for post-hoc multiple comparisons. ^&^*p* < 0.05 vs. the sham group; *p < 0.05 vs. the IR group; ^#^*p* < 0.05 vs. the oe-GDF11 + IR group. GDF11, growth differentiation factor 11; IR ischemia/reperfusion, TiL tibia length, AAR area at risk, IS infarct size.

IR mice treated with oe-GDF11 had a significantly smaller IS than control IR mice, while silencing of GDF11 increased the IS (Fig. 3A–C). The four-week mortality due to IR was 18.3%, while it was 11.2% and 24.3% in oe-GDF11-treated and sh-GDF11-treated IR mice, respectively (log-rank *p* < 0.05, Fig. 3D). These results suggest that GDF11 exerts a cardioprotective effect against IR injury. Histological examination was performed 4 weeks after surgery. Compared with the NC+sham group, there is no significant difference in mice survival rates (Fig. 3D), heart weight (HW)/body weight (BW) ratio, HW/tibia length (TiL) ratio, lung weight (LW)/BW ratio, and LW/TiL ratio in oe-GDF11+sham and sh-GDF11+sham groups (Fig. 3F–I). Compared with untreated IR mice, oe-GDF11-treated IR mice had a significantly smaller infarct scar size, HW/BW ratio, HW/TiL ratio, LW/BW ratio, and LW/TiL ratio, while opposite results were obtained in sh-GDF11-treated IR mice (Fig. 3E–I). Similarly, echocardiography showed that there was no significant difference on cardiac remodeling and function between oe-GDF11/sh-GDF11 sham mice and NC+sham group. However, oe-GDF11-treated IR mice had significantly smaller left ventricular dimensions, a higher left ventricular ejection fraction and higher fractional shortening, while sh-GDF11-treated mice had larger left ventricular dimensions and a lower left ventricular ejection fraction and lower fractional shortening (Fig. S5A–E). These findings indicated that change of myocardial GDF11 expression level in mice under normal condition exerts no influence on survival rate and cardiac morphology and function, but oe-GDF11 prevents or reverses cardiac I/R induced remodeling and heart failure.

### GDF11 alleviates mitochondrial damage induced by IR

**Fig. 4:**
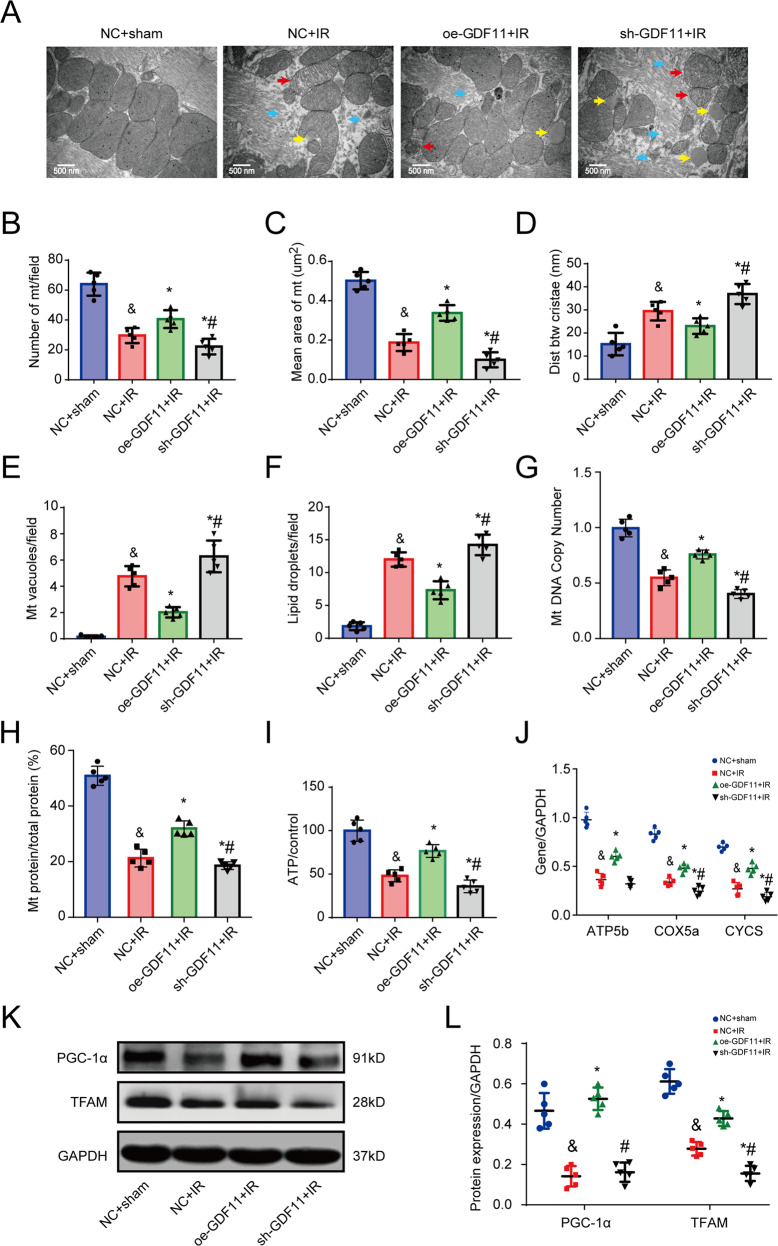
Effects of overexpressing or silencing GDF11 on mitochondrial damage induced by IR in mice. **A** Representative pictures of the ultrastructure of myocardial tissue under a transmission electron microscope. Yellow arrows: fat droplets; red arrows: vacuolated mitochondria (mt); blue arrows: broken myocardial fibril. **B** The number of mitochondria per field. **C** The mean area of a mitochondrion. **D** The distance between (btw) mitochondrial cristae. **E** The number of vacuoles per field. **F** The number of lipid droplets per field. **G** The mitochondrial DNA (mtDNA) copy number. **H** The proportion of mitochondrial protein relative to total myocardial protein. **I** ATP production. **J** mRNA expression of ATP5b, CYCS, and COX5a. **K** Representative images of western blots for PGC-1α and TFAM. **L** Semiquantitative analysis of the protein expression of PGC-1α and TFAM. ^&^*p* < 0.05 vs. the sham group; *p < 0.05 vs. the IR group; ^#^*p* < 0.05 vs. the oe-GDF11 + IR group; *n* = 5, one-way ANOVA followed by Bonferroni’s correction for post hoc multiple comparisons in (**B**–**I**) and (**K**). GDF11 growth differentiation factor 11, IR ischemia/reperfusion, oe-GDF11 GDF11 overexpression, sh-GDF11 short hairpin RNA of GDF11.

In contrast to the dense and tight mitochondria and neatly arranged and intact myocardial fibrils in the sham group, IR mice exhibited fewer mitochondria as well as myocardial fibril rupture or disappearance and manifestations of mitochondrial injury, including relaxation and swelling of mitochondria; rupture of the mitochondrial outer membrane; increases in mitochondrial cristae distance and the number of vacuolation; and lipid droplets. However, this damage was significantly attenuated by treatment with oe-GDF11 and exacerbated by sh-GDF11 (Fig. 4A–F).

We further evaluated mtDNA copy number, mitochondrial protein content, and ATP production. There were significant decreases in mtDNA copy number, mitochondrial protein content and ATP production in the IR group compared with the sham group. However, mtDNA copy number, mitochondrial protein content, and ATP production were higher in the oe-GDF11-treated group than the IR group, but lower in the sh-GDF11-treated group (Fig. 4G–I).

We also examined several key factors involved in oxidative phosphorylation, namely, ATP synthase, cytochrome C, and cytochrome oxidase. The mRNA expression levels of ATP synthase beta subunit (ATP5b), cytochrome c somatic (CYCS), and cytochrome c oxidase subunit 5A (COX5a) were significantly downregulated in response to myocardial IR, and this effect was blocked by oe-GDF11 treatment and enhanced by sh-GDF11 treatment (Fig. 4J). The protein expression of PGC-1α and TFAM was significantly lower in IR mice than in sham-operated mice, while oe-GDF11 increased and sh-GDF11 decreased the expression of PGC-1α and TFAM (Fig. 4K–L).

### GDF11 suppresses IR-induced myocyte apoptosis

**Fig. 5:**
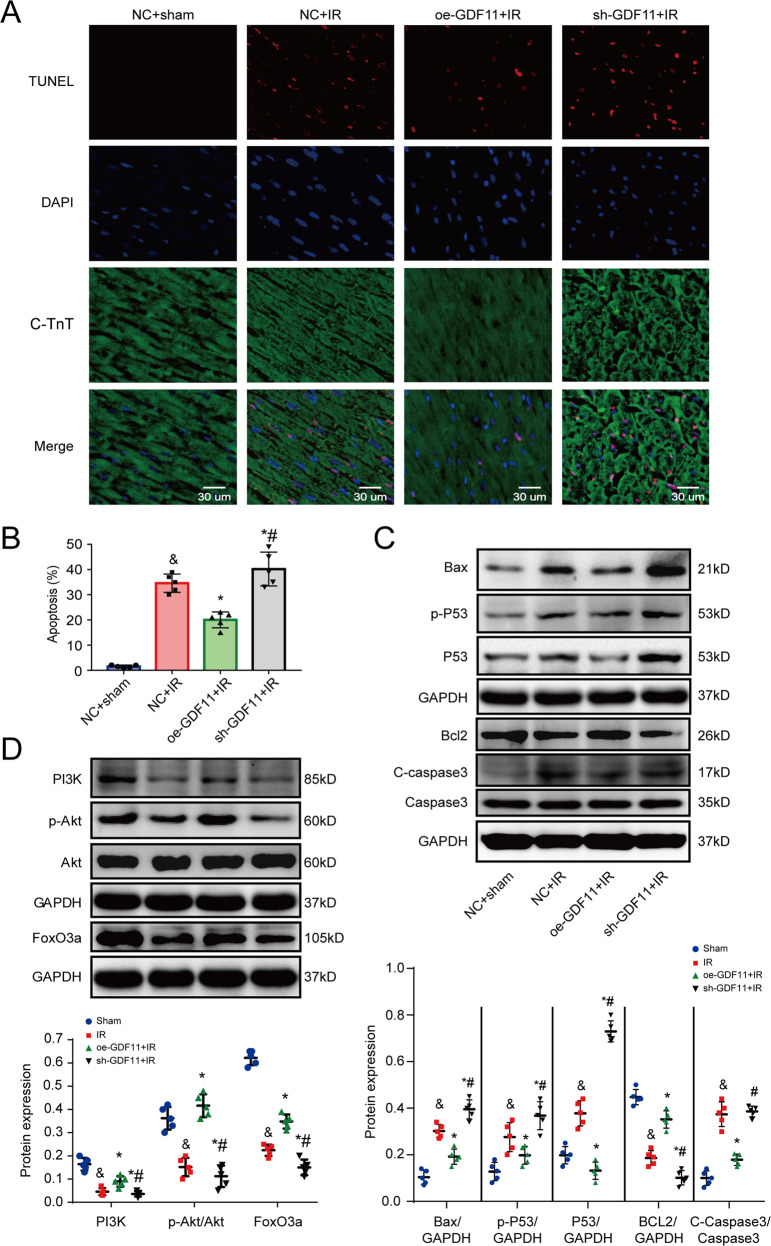
Effects of overexpressing or silencing GDF11 on IR-induced myocyte apoptosis in mice. **A** Representative photographs of TUNEL-stained cells. **B** Quantitative analysis of TUNEL-positive cells. **C** Representative images and semiquantitative analysis of western blots for Bax, P53, p-P53, C-caspase3, Caspase3, and Bcl2. **D** Representative images and semiquantitative analysis of western blots for PI3K, Akt, FoxO3a, and p-Akt. ^&^*p* < 0.05 vs. the sham group; *p < 0.05 vs. the IR group; ^#^*p* < 0.05 vs. the oe-GDF11 + IR group; *n* = 5, one-way ANOVA followed by Bonferroni’s correction for post-hoc multiple comparisons in panel (**B**, **D**, **F**). GDF11 growth differentiation factor 11, IR ischemia/reperfusion, oe-GDF11 GDF11 overexpression, sh-GDF11 short hairpin of GDF11.

It has been reported that a decrease in mitochondrial number and mitochondrial dysfunction usually aggravate apoptosis [32]. Representative photographs of TUNEL staining are shown in Fig. 5A. There were significantly more total TUNEL-positive cardiomyocyte nuclei in the IR group than in the sham group (35.63 ± 4.71% vs. 1.05 ± 0.24%, *p* < 0.01), while oe-GDF11 and sh-GDF11 suppressed and promoted apoptosis, respectively (21.10 ± 3.03% and 40.20 ± 5.23%) (Fig. 5B). The expressions of the proapoptotic proteins Bax, P53, and C-caspase3 in the mouse heart were upregulated in the IR group compared with the sham group, while treatment with oe-GDF11 and sh-GDF11 decreased and increased the expressions of Bax, P53, and C-caspase3, respectively (Fig. 5C). Similarly, the antiapoptotic pathway PI3K/Akt/FoxO3a and antiapoptotic protein Bcl2 (B-cell lymphoma 2) were inhibited in response to IR injury, while oe-GDF11 treatment significantly attenuated and sh-GDF11 treatment significantly enhanced the inhibitory effect of IR on the protein expressions of Bcl2, PI3K, FoxO3a, and the phosphorylation of Akt (Fig. 5C, D).

### GDF11 activates telomere reverse transcriptase and inhibits telomere shortening

**Fig. 6:**
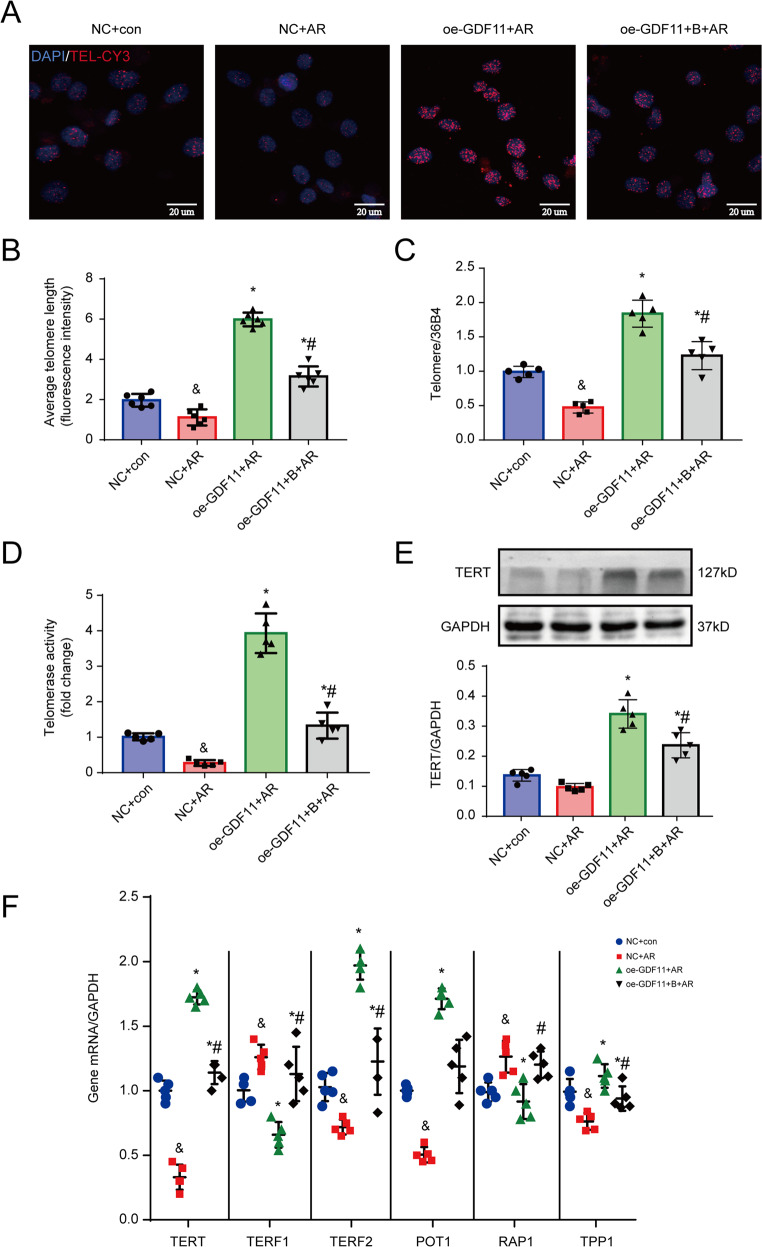
GDF11 activated telomerase and inhibited telomere shortening in NRCMs under AR. **A** Representative photographs of telomeres measured by Q-FISH in NRCMs. **B** Quantitative analysis of the TL of TEL-CY3-positive cells. **C** TL measured by real-time PCR. **D** The activity of telomerase. **E** Representative western blot images and semiquantitative analysis of TERT. **F** mRNA expression of TERT, TERF2, POT1, TPP1, TERF1, and RAP1. ^&^*p* < 0.05 vs. the NC group; *p < 0.05 vs. the AR group; ^#^*p* < 0.05 vs. the oe-GDF + AR group; *n* = 6 in each group in panel (**B**) and *n* = 5 per group in panels (**C**–**G**); one-way ANOVA followed by Bonferroni’s correction for post hoc multiple comparisons in panel (**B**–**D**, **F**, **G**). (**B**) The telomerase inhibitor BIBR1532; GDF11 growth differentiation factor 11, oe-GDF11 GDF11 overexpression, TL telomere length.

To determine whether the antiapoptotic effect of GDF11 was attributable to the inhibition of telomere shortening, we measured the TL of cultured NRCMs in response to AR and treatment with GDF11 (Fig. 6A–C). We observed that the TL was shorter in AR-treated cardiomyocytes than in cells cultured under normoxic conditions. However, oe-GDF11 alleviated the telomere shortening caused by AR, and this effect was blocked by cotreatment with the telomerase inhibitor BIBR1532 (Fig. 6B, C). Considering that telomerase controls the length of telomeres, we examined the activity of telomerase. In AR-treated cardiomyocytes, telomerase activity was significantly lower than in normoxia-treated cells, while oe-GDF11 and AR-treated cells had significantly higher telomerase activity than AR-treated cells not administered oe-GDF11, and this effect was partially offset by cotreatment with BIBR1532 (Fig. 6D).

The TERT protein expression was low in the control cells and AR stimulation further downregulated TERT expression. However, oe-GDF11 activated TERT expression, which the effect was partially antagonized by the telomerase inhibitor BIBR1532 (Fig. 6E). In addition, we found that BIBR1532 alone inhibited the expression of TERT rather than GDF11 under both normal and AR conditions (Fig. S6A, B), suggesting that GDF11 is an upstream of TERT. AR stimulation significantly downregulated the levels of TERT and telomeric repeat-binding factor 2 (TERF2), protection of telomere 1 (POT1) and tripeptidyl peptidase 1 (TPP1), but significantly upregulated the levels of telomeric repeat-binding factor 1 (TERF1) and DNA-binding transcription factor (RAP1), and these changes were alleviated by cotreatment with oe-GDF11 (Fig. 6F). Addition of BIBR1532 partially inhibited the effects of oe-GDF11 on the levels of the telomere-related genes (Fig. 6F).

### GDF11 attenuates AR-induced mitochondrial damage and suppresses myocyte apoptosis in cultured cardiomyocytes

**Fig. 7:**
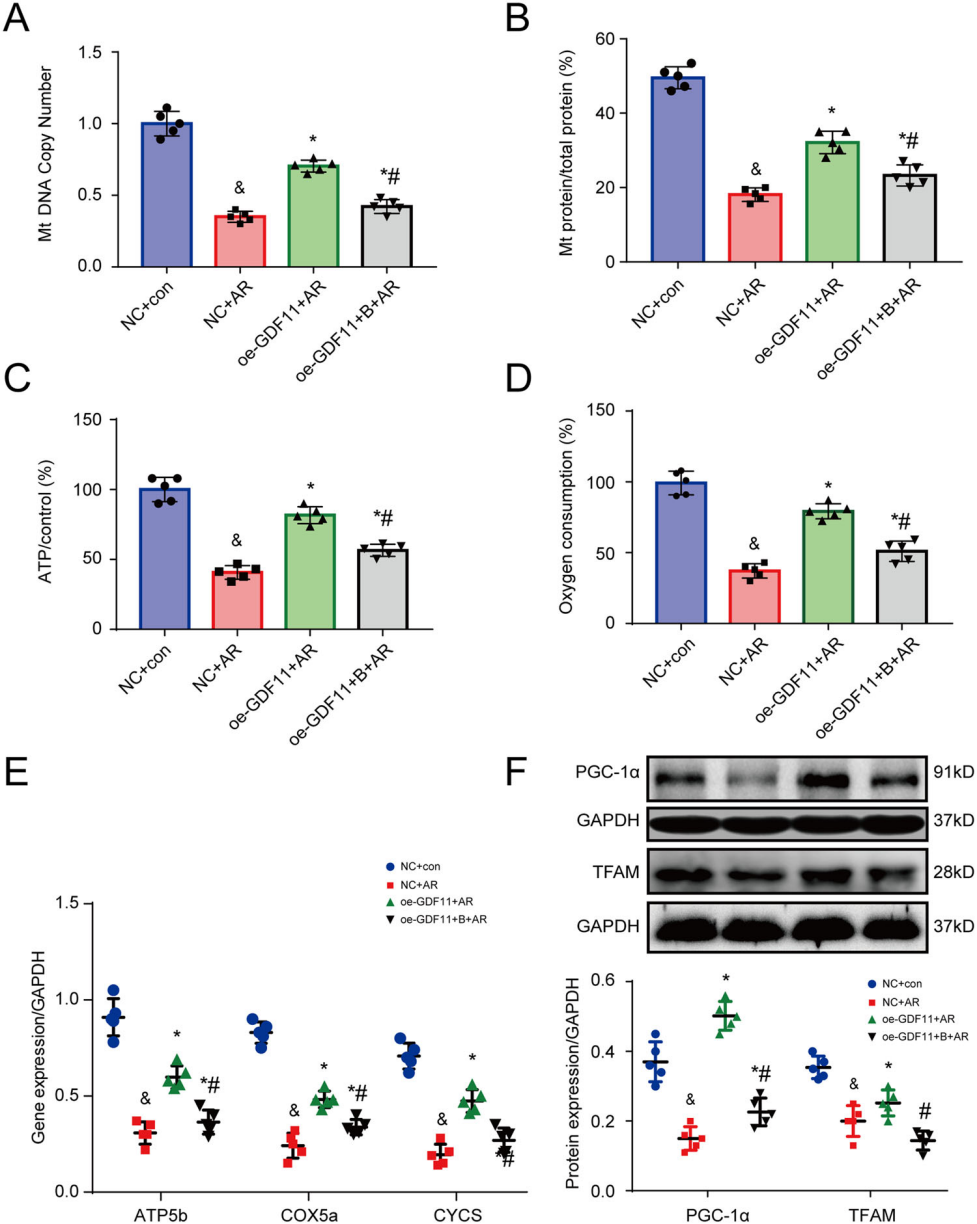
GDF11 inhibited AR-induced mitochondrial damage in NRCMs. **A** The mitochondrial (mt) DNA copy number. **B** The proportion of mitochondrial protein relative to total cell protein. **C** ATP production. **D** oxygen consumption. **E** mRNA expression of ATP5b, CYCS, and COX5a. **F** Representative western blot images and semiquantitative analysis of PGC-1α and TFAM. ^&^*p* < 0.05 vs. the NC group; *p < 0.05 vs. the IR group; ^#^*p* < 0.05 vs. the oe-GDF11 + AR group; *n* = 5, one-way ANOVA followed by Bonferroni’s correction for post-hoc multiple comparisons in panel (**A**–**C**, **E**). AR anoxia/reoxygenation; B the telomerase inhibitor BIBR1532, GDF11 growth differentiation factor 11, oe-GDF11 GDF11 overexpression.

In cultured NRCMs, we examined the effects of oe-GDF11 on mtDNA copy number, mitochondrial protein content, ATP production, oxygen consumption, the mRNA levels of ATP synthase beta subunit (ATP5b), cytochrome c somatic (CYCS), and cytochrome c oxidase subunit 5A (COX5a), and the protein expression of PGC-1α and TFAM. Compared to control cells cultured under normoxic conditions, cells subjected to AR exhibited significant decreases in mtDNA copy number, mitochondrial protein content, ATP production, and oxygen consumption, significant downregulation of the mRNA levels of ATP5b, CYCS and COX5a, and significant downregulation of the protein levels of PGC-1α and TFAM. These effects were partially reversed by cotreatment with oe-GDF11 (Fig. 7A–F). The beneficial effects of oe-GDF11 were antagonized by the telomerase inhibitor BIBR1532 (Fig. 7A–F).

Similar to the results obtained in the mouse model of IR, AR increased apoptosis, upregulated the related proapoptotic proteins Bax, P53, p-P53, C-caspase3, and inhibited the antiapoptotic pathway PI3K/Akt/FoxO3a and antiapoptotic Bcl2 in NRCMs, and these effects were blocked by cotreatment with oe-GDF11 (Fig. S7A–G). The beneficial effects of oe-GDF11 were antagonized by the telomerase inhibitor BIBR1532 (Fig. S7A–G).

## Discussion

**Fig. 8:**
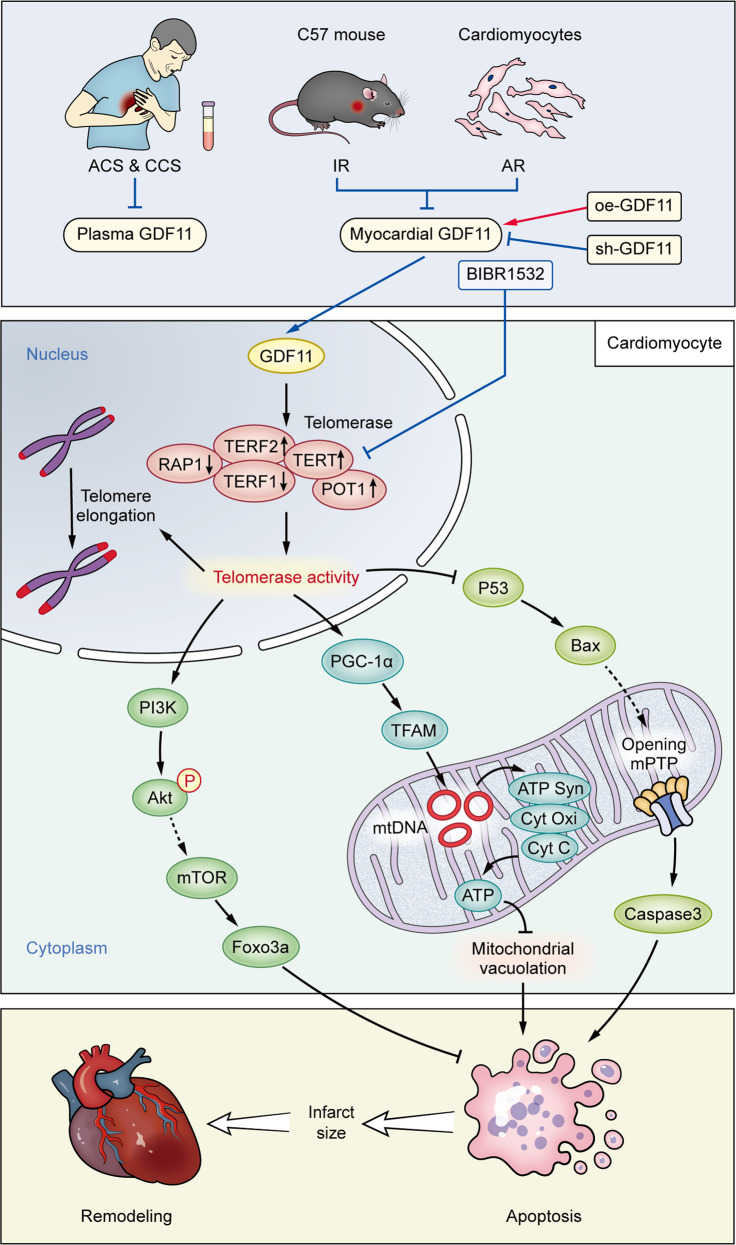
An illustration of the influences of GDF11 on IR/AR injury and the underlying mechanisms. Patients with CAD, especially ACS, and rodent cardiomyocytes subjected to IR or AR insult, exhibit lower circulatory or myocardial GDF11 levels, leading to lower activity of telomerase and shorter TL in the nucleus due to downregulation of TERT, inhibiting mitochondrial energy dynamics and the antiapoptotic PI3K/Akt/FoxO3a pathway and activating the proapoptotic p53/Bax pathway. These changes collectively increase cardiomyocyte apoptosis, enlarge the myocardial infarct size and eventually promote cardiac remodeling. The changes can be antagonized and enhanced by overexpression and silencing of GDF11, respectively, and the beneficial effects of GDF11 can be attenuated by the telomerase inhibitor BIBR1532. ↑: activation, ⊥: inhibition.┆: from other reference.│: experimentally proven.

Our main findings in this study can be summarized as follows: (1) Circulating GDF11 levels in patients with ACS were significantly lower than those in patients with CCS; (2) Endogenous GDF11 attenuated mitochondrial damage in cardiomyocytes subjected to IR or AR through improving the energy dynamics of mitochondria; (3) GDF11 limited the myocardial IS in the early stage and inhibited IR-induced cardiac remodeling in the late stage; (4) GDF11 prevented AR-induced TL shortening by increasing telomerase activity in cardiomyocytes. An illustration of the influences of GDF11 on IR/AR injury and the underlying mechanisms is shown in Fig. 8.

GDF11 has been demonstrated to act as a rejuvenating factor, and several studies have shown that GDF11 can restore the function of multiple organs in old mice [4, 6, 33]. Although some reports have questioned the purported age-related decrease in circulating GDF11 levels [34, 35], the clinical data in this study provide new evidence that plasma GDF11 concentrations are negatively correlated with age in healthy controls. In contrast to the conflicting findings on the role of GDF11 in cardiac hypertrophy [35], a few studies with consistent results have demonstrated that GDF11 exerts a protective effect against myocardial ischemia [12, 36]. An interesting finding of this study is that circulating GDF11 levels were significantly lower in ACS patients than in CCS patients, suggesting that the role of GDF11 in the heart is dependent on the degree of ischemia.

Regarding the role of GDF11 in chronic cardiac remodeling after ischemia, Olson et al. reported that higher GDF11/8 levels are associated with a lower incidence of cardiac hypertrophy and a lower risk of cardiovascular events and death in patients with stable ischemic heart disease, and similar results have been reported in mice by Du et al., who showed that exogenous GDF11 alleviates chronic heart failure after acute IR injury [12]. Consistently, by overexpressing the GDF11 protein by about 2-fold, we demonstrated that GDF11 attenuated post-IR remodeling. However, the underlying mechanisms have not been fully clarified.

In this study, we focused on the mechanisms by which GDF11 protects against IR. A previous study reported that the ability of GDF11 to restore the function of the aged mouse heart and alleviate chronic heart failure is associated with c-kit positive myocardial stem cell regeneration after acute IR injury [12], but undeniable evidence has shown that the adult mammalian heart lacks an endogenous regenerative stem cell labeled c-kit [37, 38]. It has been reported that reductions in oxidation and inflammation resulting from attenuation of noncanonical TGF-β signaling also contribute to the attenuation of IR injury by GDF11 [36]. Considering the important role of mitochondrial function and TL in aging and myocardial ischemia [17, 39, 40], it is reasonable to predict that GDF11, a novel “rejuvenation” factor capable of relieving IR injury and retarding the aging process, may exert cardioprotective effects by influencing mitochondrial function and TL.

Currently, only a few lines of evidence showing that GDF11 improves mitochondrial function are available in PubMed. An investigation by Zhao et al. indicated that GDF11 enhances the therapeutic effects of cardiac mesenchymal stem cells on the infarcted myocardium by promoting mitochondrial fusion, and preserving mitochondrial morphology and function under hypoxic conditions [41]. Xiao et al. reported that exogenous GDF11 alleviates intracerebral hemorrhage-induced apoptosis, oxidative stress, and mitochondrial damage in the perihematomal tissues of mice [42], while Garrido-Moreno et al. noted that GDF-11 attenuates cardiomyocyte hypertrophy by preventing the loss of communication between the sarcoplasmic reticulum and mitochondria [43]. Mitochondria are highly dynamic organelles that adapt to various stress conditions through continuous fusion, and division to meet the energy metabolism and other biological needs of the cell. This biological process is called mitochondrial dynamics [44]. The relationship between mitochondrial dynamics and energy demand, suggesting changes in mitochondrial architecture as a mechanism for bioenergetic adaptation to metabolic demands. Mitochondrial energetic dynamics refers to the balancing of mitochondrial dynamics and energetics to maintain mitochondrial homeostasis [45]. In our study, several aspects of mitochondrial energy dynamics were evaluated. We found that overexpression of GDF11 blocked IR-induced loss and destruction of mitochondria, as evidenced by higher mtDNA copy number, mitochondrial protein content, and ATP production in oe-GDF11-treated IR mice than in untreated IR mice. As expected, oe-GDF11 upregulated several key factors involved in oxidative phosphorylation, including the mRNA levels of ATP5b, CYCS, and COX5a, and the mitochondria protection-related proteins PGC-1α and TFAM. The alleviation of mitochondrial damage by GDF11 is likely partially attributable to less apoptosis and eventually a smaller IS and less cardiac remodeling.

Why does GDF11 improve mitochondrial energy dynamics in cardiomyocytes following IR or AR injury? There is a close association between mitochondria and telomeres, and both play an important roles in cardiovascular disease and cellular senescence. Previous reports have demonstrated that a shorter leukocyte TL is associated with a worse clinical outcome in patients with ischemic heart failure, that patients with previous myocardial infarction have a 20% shorter leukocyte TL than patients without myocardial infarction, and that both leukocyte TL and GDF11 are inversely correlated with age [46]. Cataldi et al. showed that anoxia can produce oxidative stress, resulting in telomere shortening in cardiomyocytes [47, 48]. Telomeres are located in the nucleus, which is also a subcellular compartment of GDF11 [49]. Our immunofluorescence staining images of GDF11 in NRCMs showed that GDF11 is distributed in cytoplasm, nucleus and extracellular space (Fig. S8). As a secretory protein, GDF11 can bound to the extracellular matrix just like many growth factors [50]. In addition, it is reported that GDF11 is transported and stored in vesicles such as lipid droplets, endosomes, lysosomes and peroxisomes [49], and it is possible to transport GDF11 to the nuclear plasma from the transport vesicles [51]. Besides, recent observations have indicated that telomerase is also localized in cytoplasm and mitochondria [52], suggesting that cytoplasmic GDF11 may interact with it in cytoplasm and mitochondria. However, there have been no reports on the relation between telomeres and GDF11. We found that cardiomyocytes subjected to AR exhibited shorter telomeres and lower telomerase activity than cells cultured under normoxia and that these damaging effects were partially blocked by GDF11. Furthermore, the beneficial effects of GDF11 on mitochondrial function were antagonized by a telomerase inhibitor. Our findings suggest that GDF11 can improve mitochondrial function by preventing ischemia-induced decreases in telomerase activity and TL shortening.

It is known that both mitochondria and telomeres play critical roles in inhibiting apoptosis [14]. We noted that in addition to inhibiting apoptosis by alleviating mitochondrial damage, GDF11 suppressed apoptosis by inhibiting the proapoptotic P53/Bax pathway [20, 53] and promoting the antiapoptotic PI3K/Akt/FoxO3a signaling pathway through activation of telomerase. Consistently, it has been reported that an increase in TL resulting from upregulation of telomerase can protect the heart from fatal damage by severe anoxia [54], while TERT represses the expression of tumor necrosis factor-related apoptotic genes, B-cell lymphoma 2 (BCL2) and P53 [55]. These data suggest that inhibition of telomerase may aggravate apoptosis by promoting the release of proapoptotic factors, and inhibiting antiapoptotic factors in both mitochondrial-dependent and mitochondrial-independent pathways.

To further verify the regulatory relationship between GDF11 and telomerase, we detected the mRNA expression of telomerase subunits. We found that TERT, TERF2, POT1, and TPP1, which may promote telomere elongation, were downregulated after AR, while the subunits TERF1 and RAP1, which may inhibit telomere elongation, were upregulated. Importantly, GDF11 overexpression upregulated TERT, TERF2, and POT1 expression and downregulated TERF1 and RAP1 expression. These data suggest that GDF11 is able to regulate different subunits of telomerase to elongate telomeres in cardiomyocytes under AR.

As a secreted cytokine in the TGF-β family, GDF11 is widely distributed in various organs [56]. There is accumulating evidence that organs such as heart, lung, or kidney secrete soluble factors, including GDF11 [57, 58]. Myocardial overexpression/knockdown of GDF11 increased/decreased plasma levels of GDF11 by approximately 1.3-fold and 0.8-fold in mice, respectively. A previous study has shown that small change in plasma GDF11 concentration in mice did not cause systemic effects [59]. Taken together, delivery of GDF11 in the heart can avoid the potential systemic adverse effects such as muscle atrophy and cachexia. High doses of GDF11 cause severe cachexia and death. The improper use of GDF11 could have potentially devastating actions on the heart and other tissues [60]. Although limited by a small sample size, data from this study support the protective effects of myocardial ischemia-reperfusion injury of GDF11 through activation of telomerase in animal models. We will further expand the sample size for multicenter trials to improve the value of clinical transformation.

In conclusion, our findings indicate that GDF11 contributes to limiting morphologic and functional damage of mitochondria in cardiomyocytes following IR or AR insult, and repressing apoptosis in mitochondria-dependent and mitochondria-independent manners by increasing telomerase activities.

## Supplementary information

Supplemental material
