# Supplementary figures and images for: Growth differentiation factor 11 attenuates cardiac ischemia reperfusion injury via enhancing mitochondrial biogenesis and telomerase activity
Source: Cell Death Dis. 2021 Jul 2;12(7):665. doi: 10.1038/s41419-021-03954-8 (PMC8253774; doi:10.1038/s41419-021-03954-8)

## Slide 1
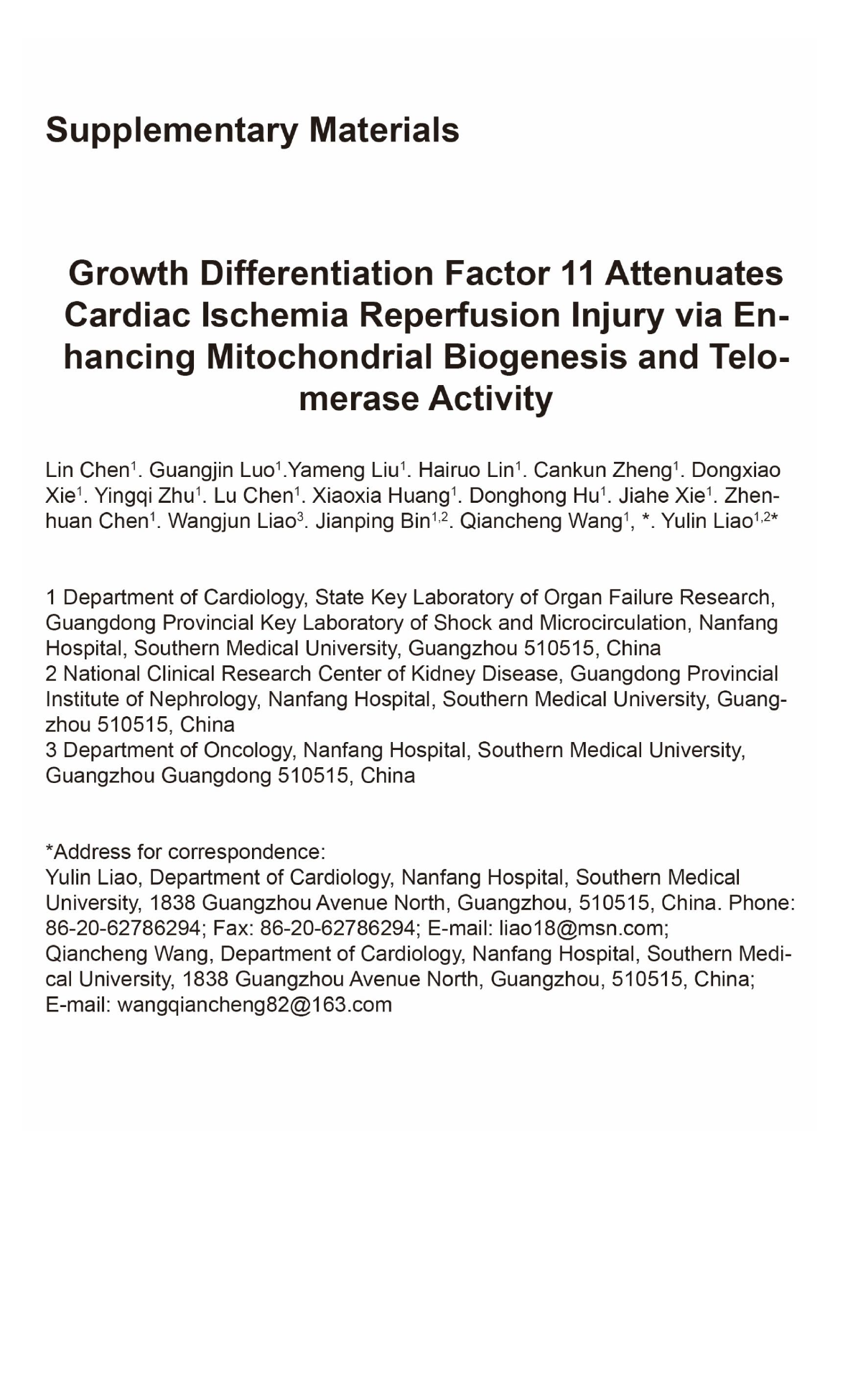

## Slide 2
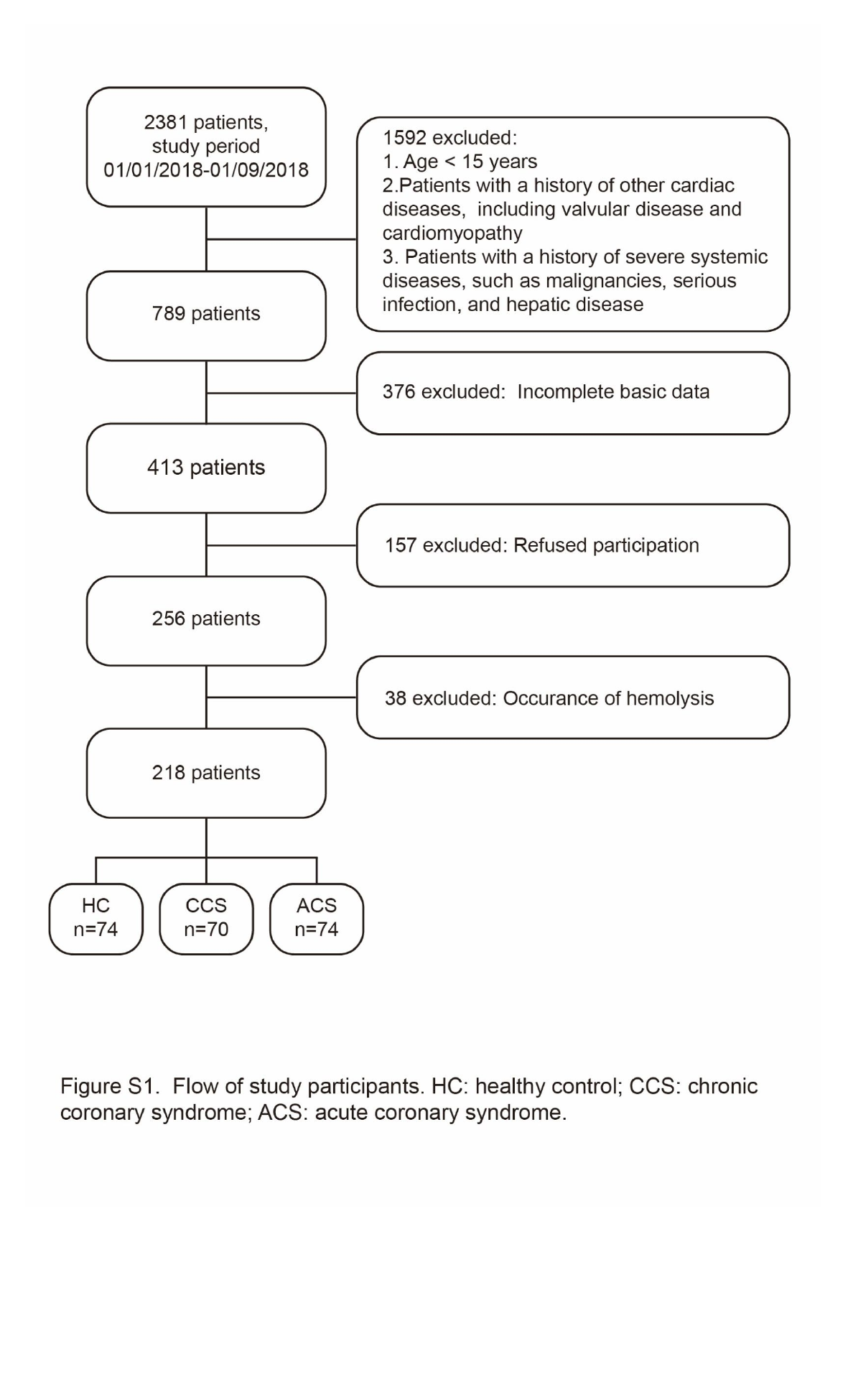

## Slide 3
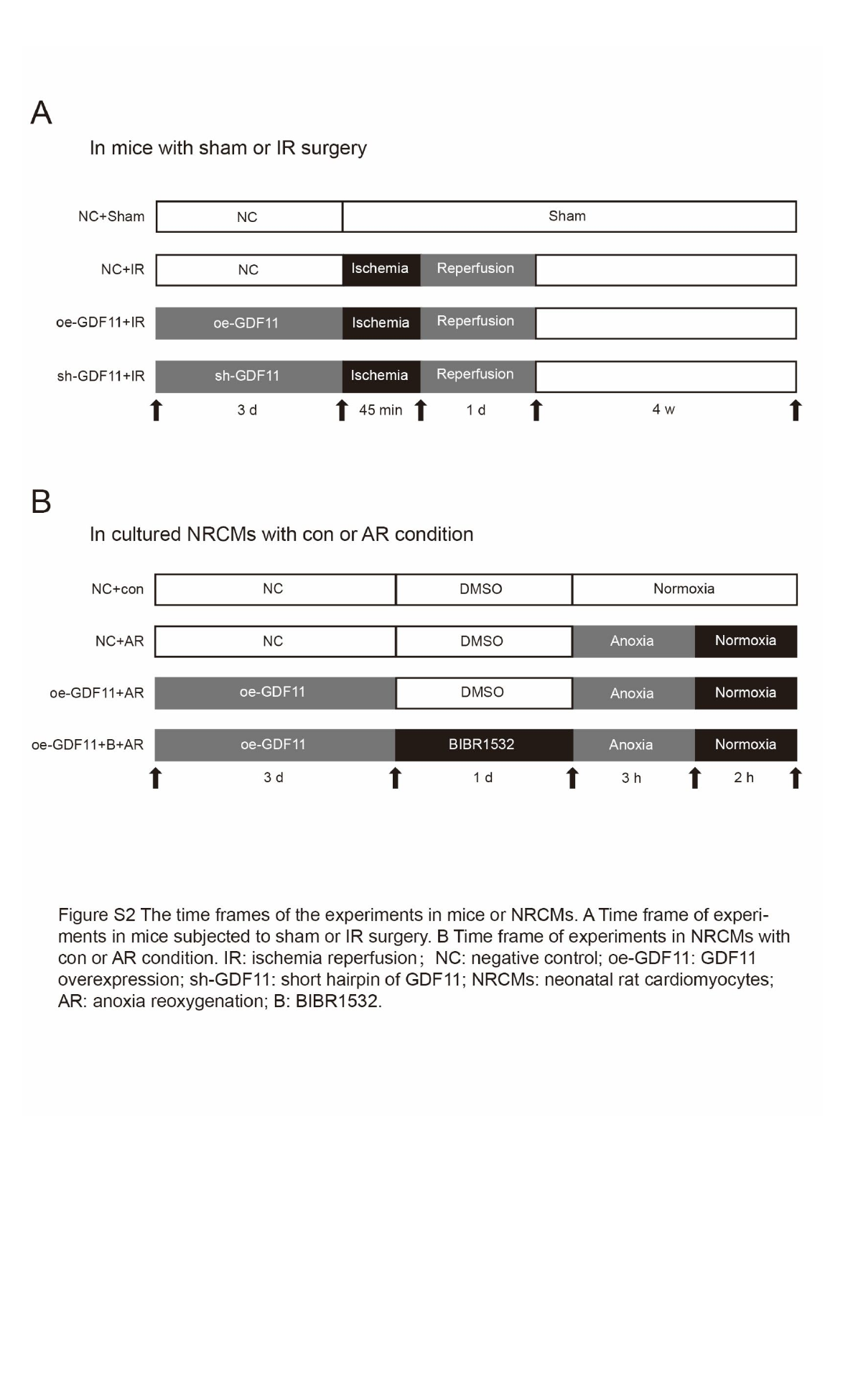

## Slide 4
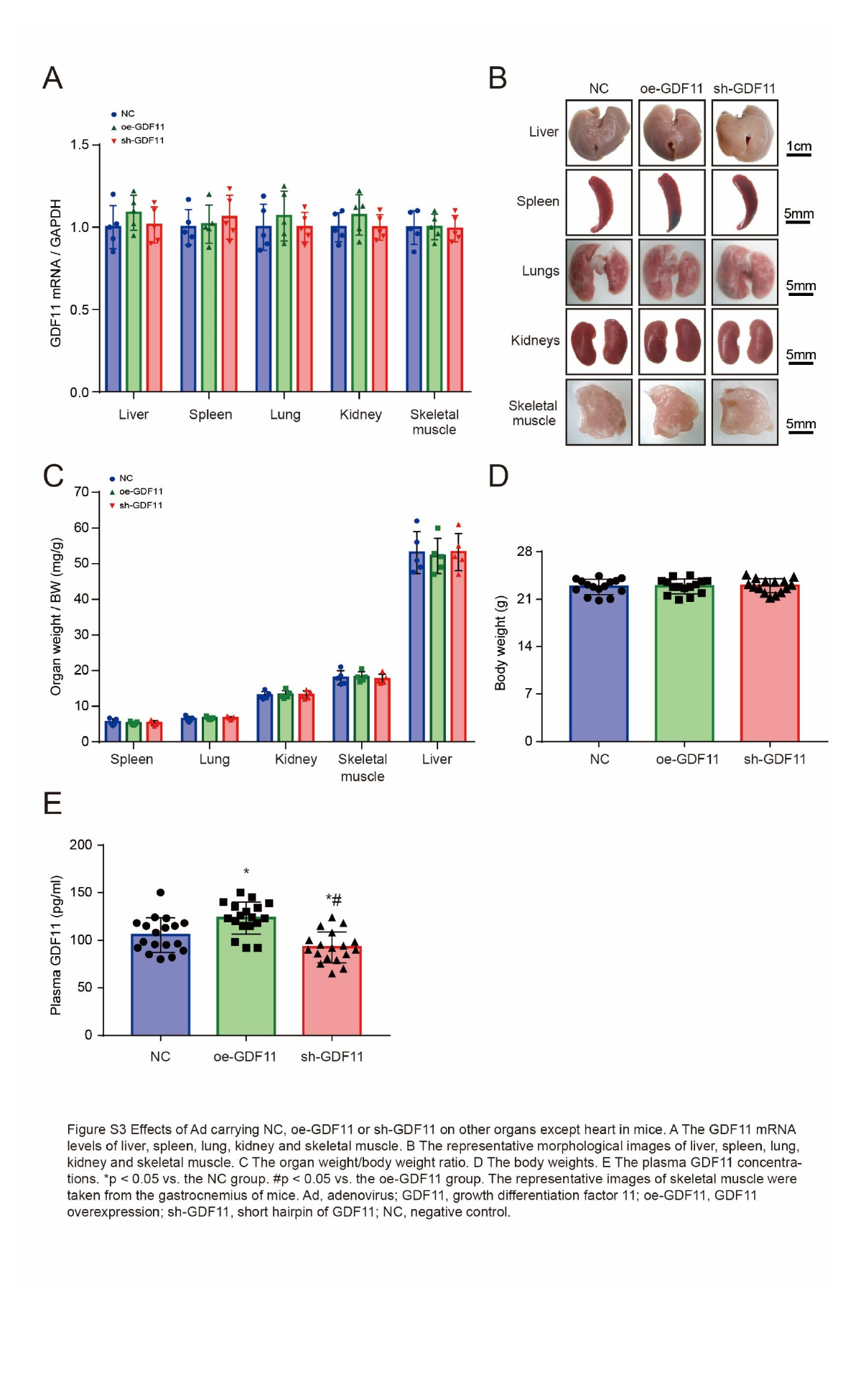

## Slide 5
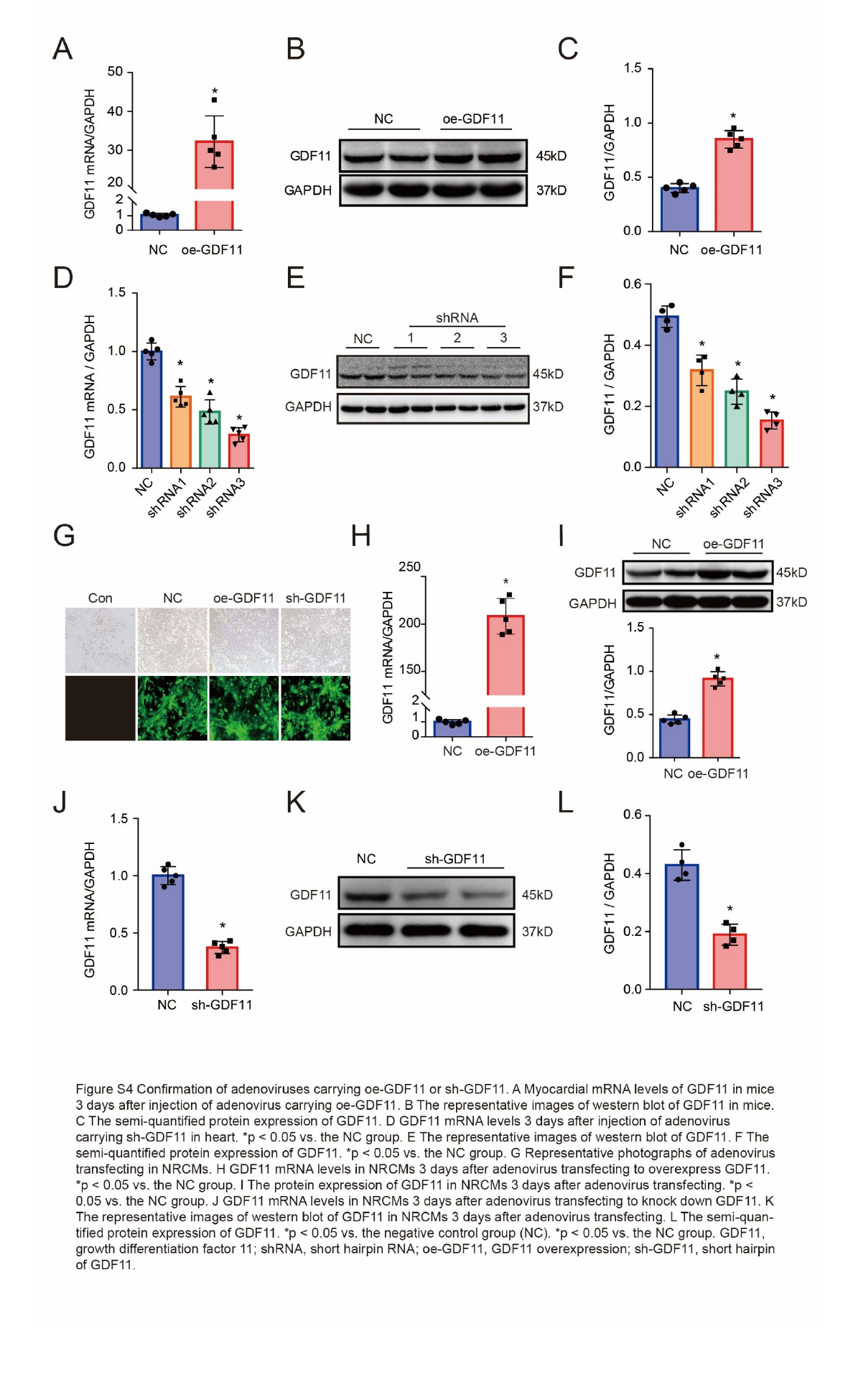

## Slide 6
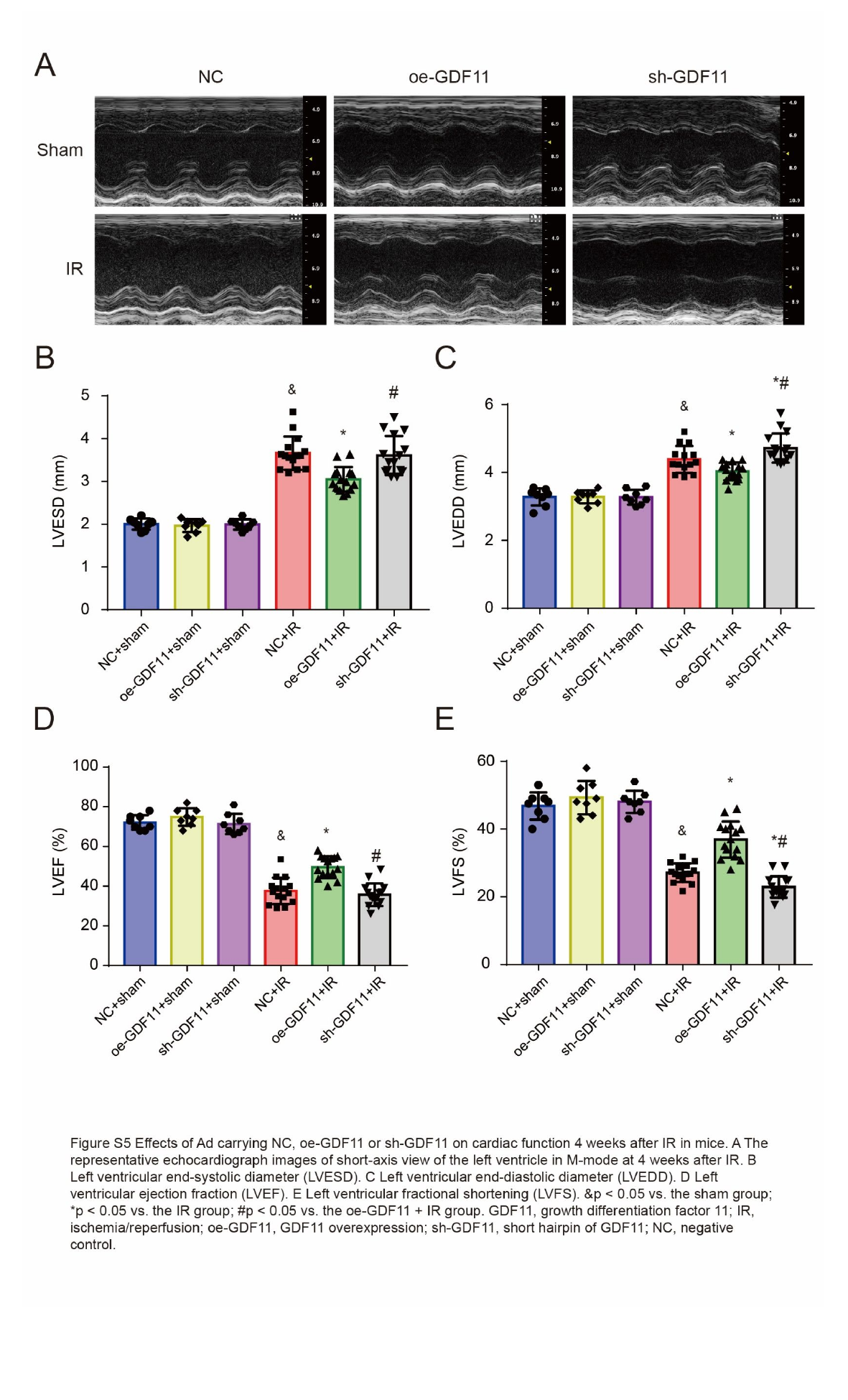

## Slide 7
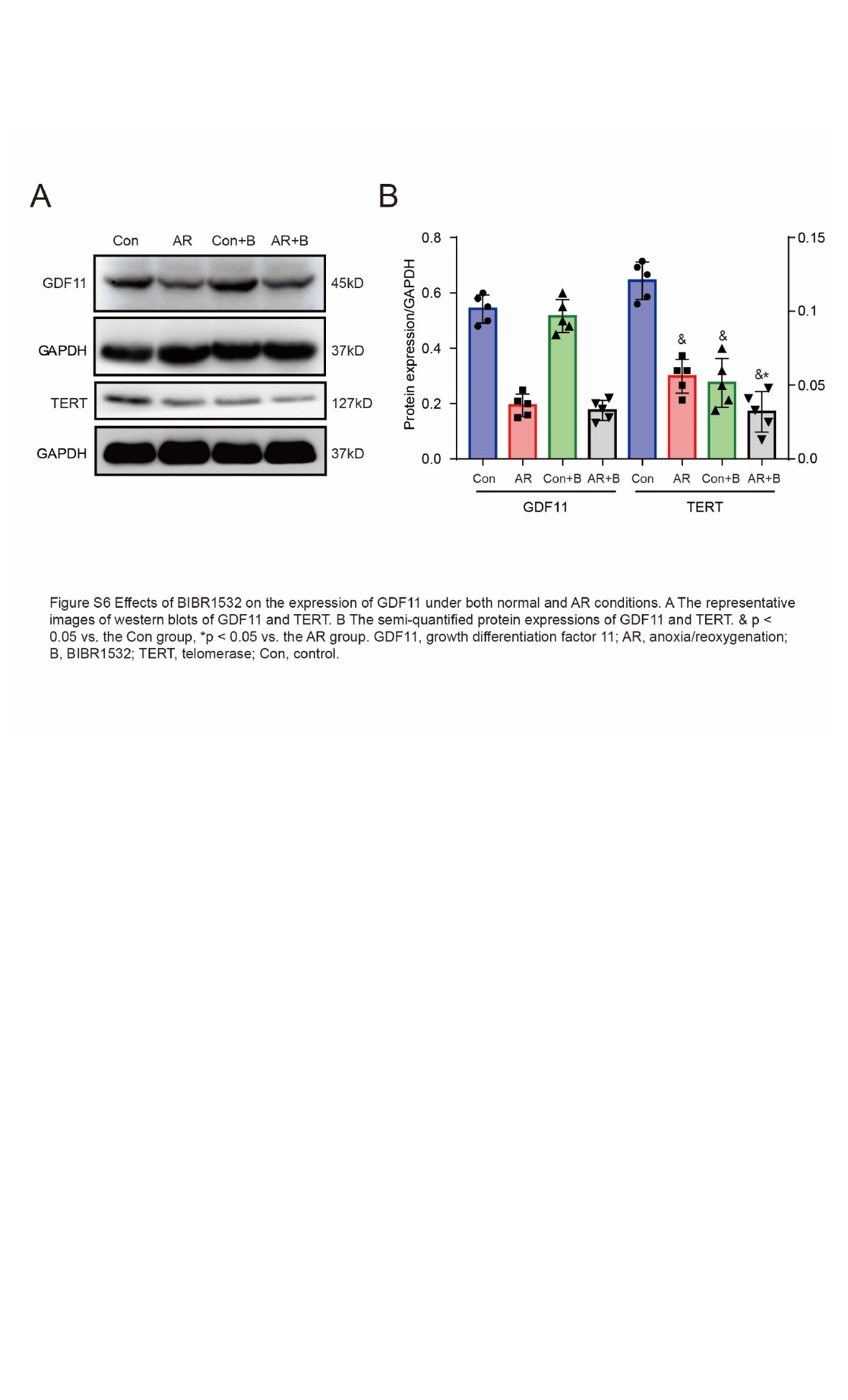

## Slide 8
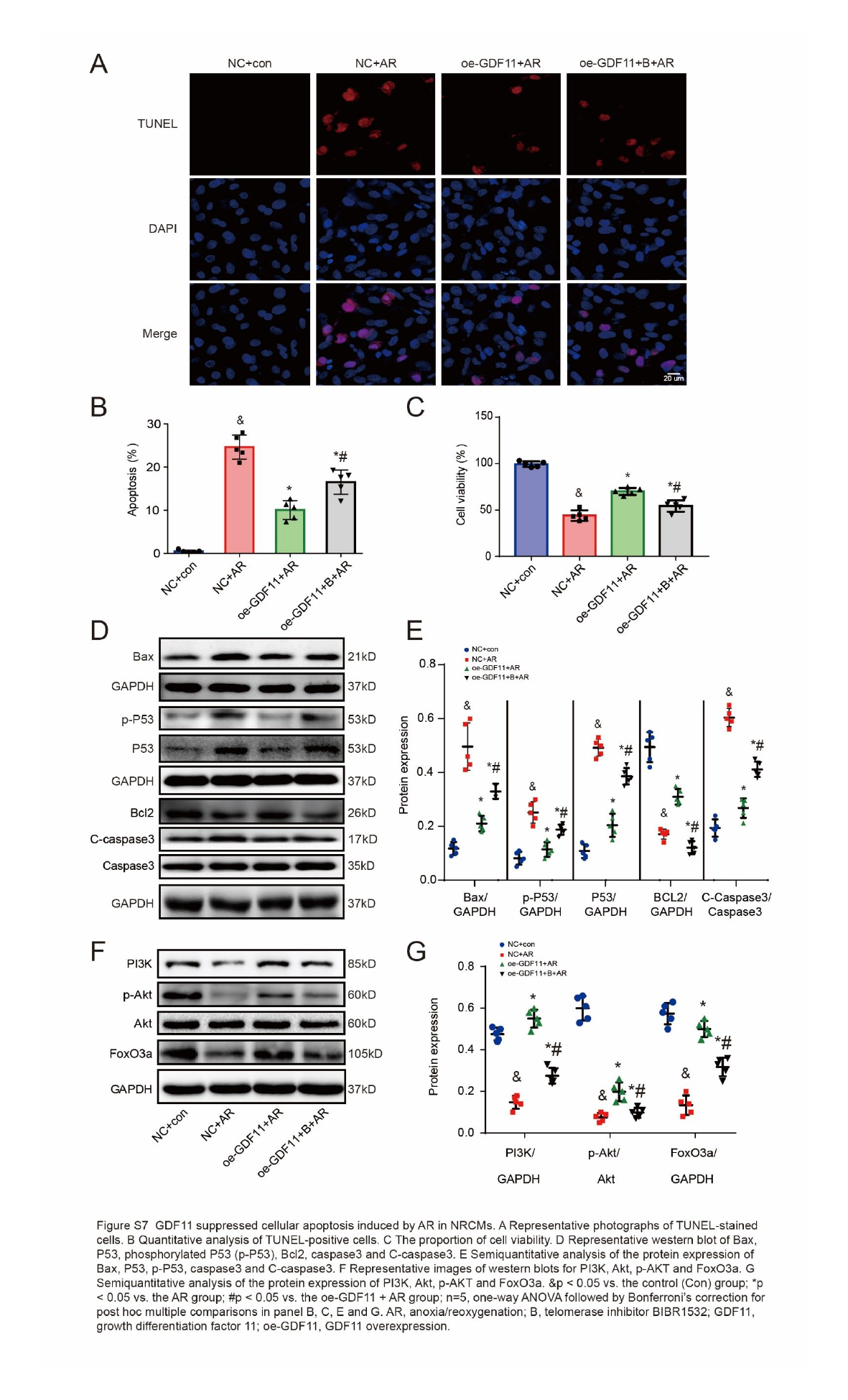

## Slide 9
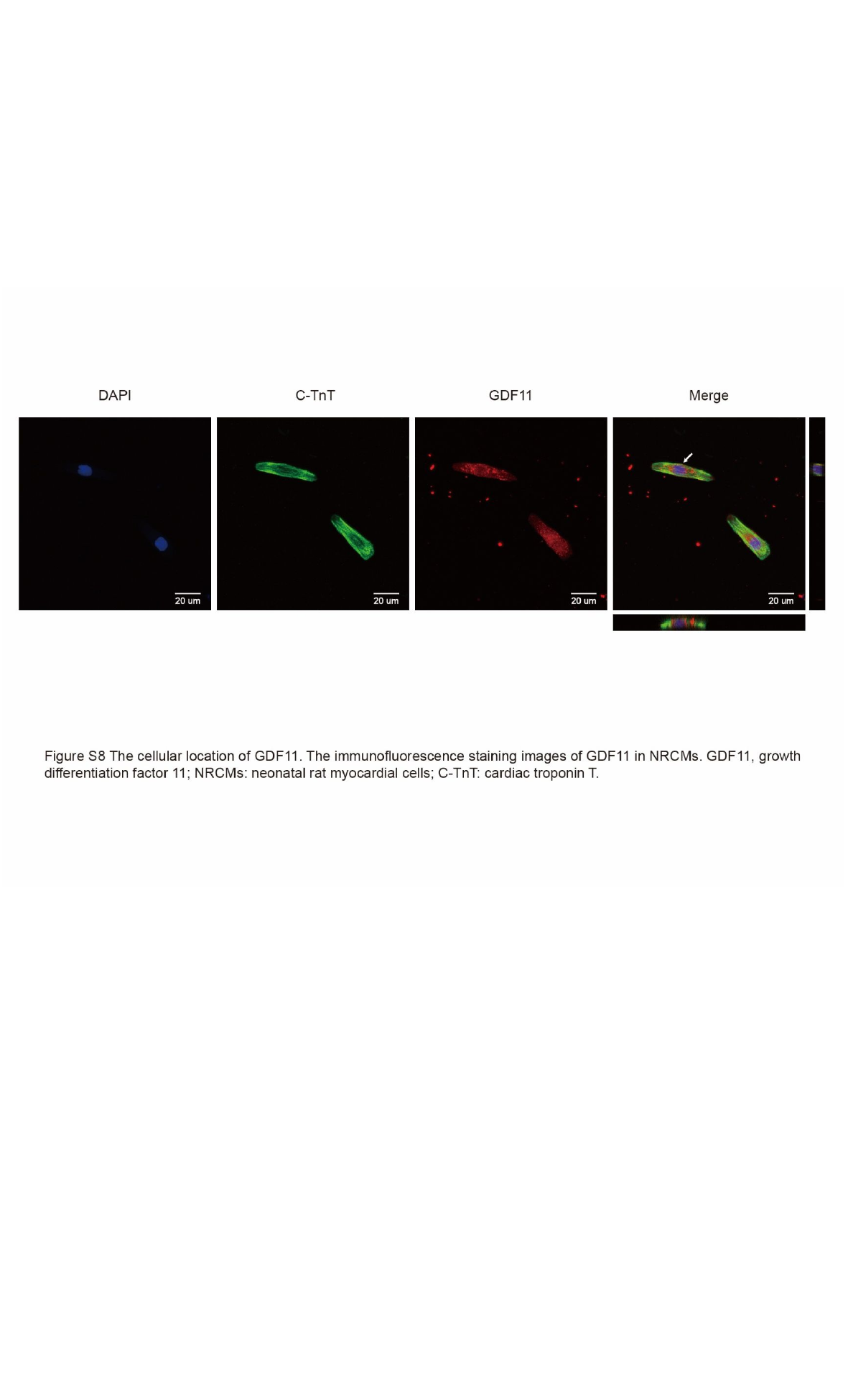

## Slide 10
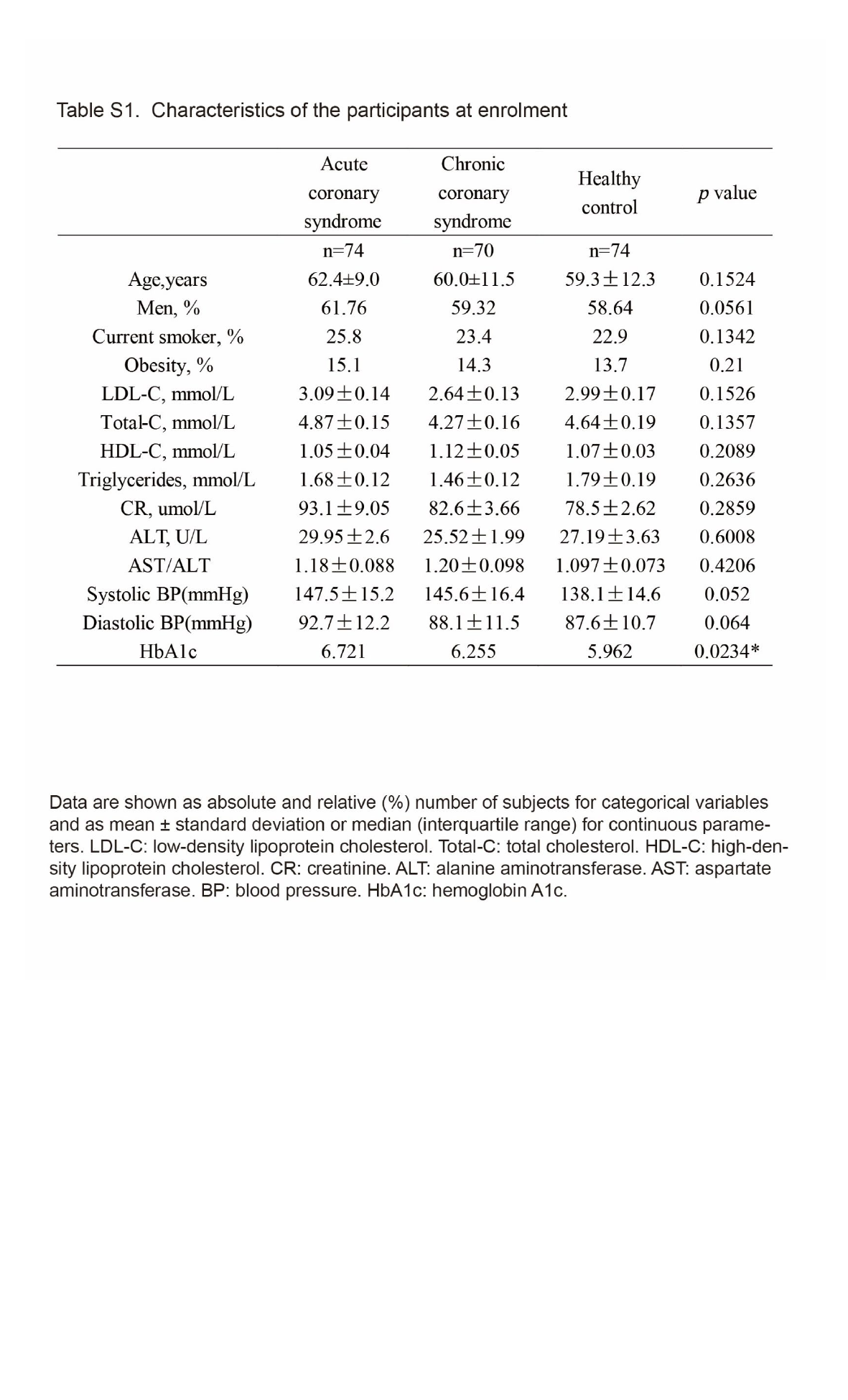

## Slide 11
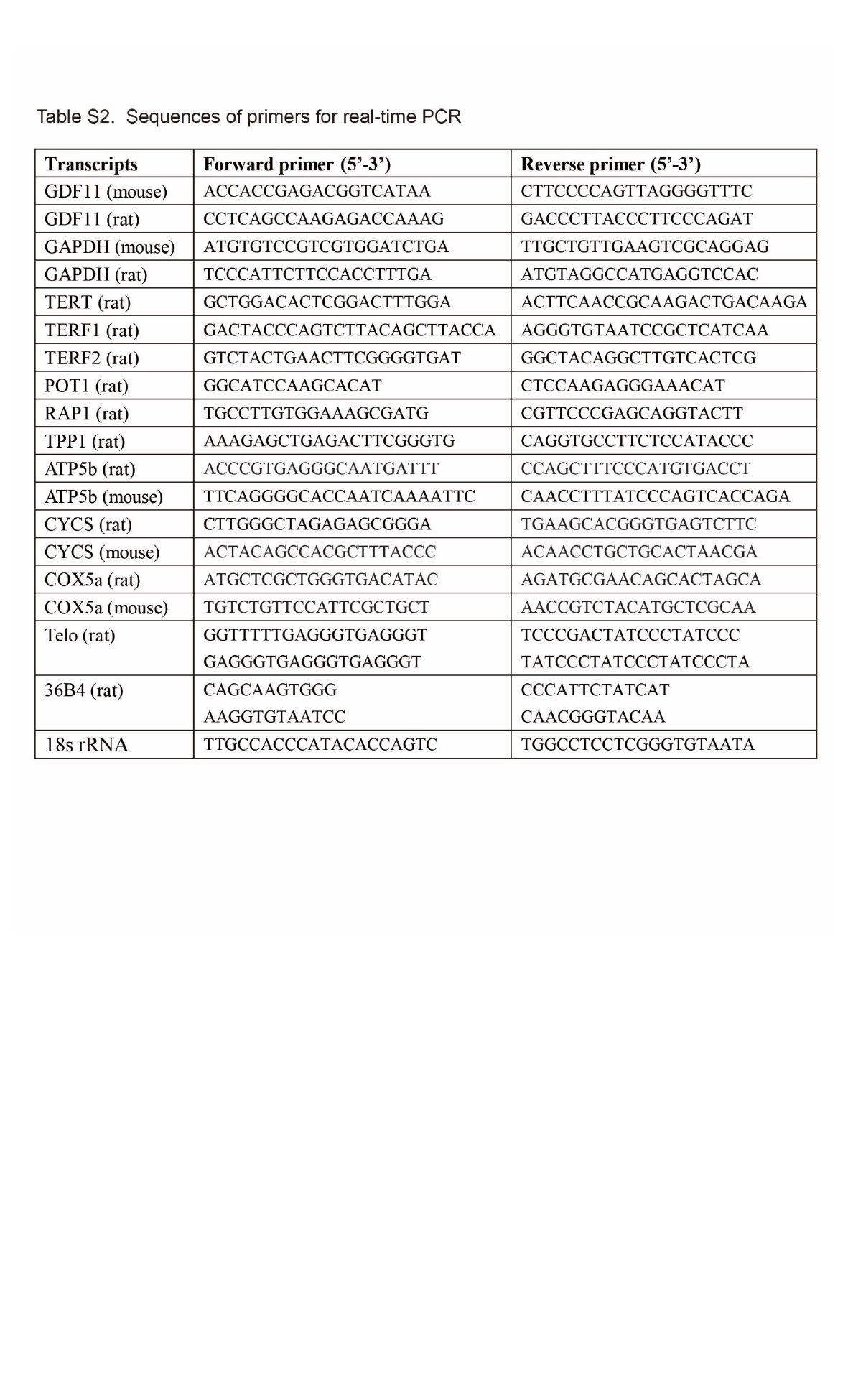

Supplement: Supplementary file 1 — Supplemental material [file 41419_2021_3954_MOESM1_ESM.pptx]
